# PTHrP induces breast cancer bone metastases via microbial-dependent expansion of TNF/IL-17–producing T cells

**DOI:** 10.1172/jci.insight.207567

**Published:** 2026-10-22

**Authors:** Nand K. Roy, Subhashis Pal, Hamid Y. Dar, Sanchiti Patil, M. Neale Weitzmann, Roberto Pacifici

**Affiliations:** 1 Division of Endocrinology, Metabolism and Lipids, Department of Medicine, and; 2 Emory Microbiome Research Center, Emory University, Atlanta, Georgia.; 3 Joseph Maxwell Cleland Atlanta VA Healthcare System, Decatur, Georgia.; 4 Immunology and Molecular Pathogenesis Program, Emory University, Atlanta, Georgia, USA.

**Keywords:** Bone biology, Immunology, Oncology, Breast cancer

## Abstract

Bone metastases are a frequent complication of breast cancer, leading to reduced quality of life and increased morbidity. By intratibially injecting PyMT breast cancer cells in mice, we showed that the presence in the gut microbiome of segmented filamentous bacteria (SFB), a taxon that induces IL-17–producing Th17 cells and TNF-α (TNF)–producing T cells (TNF^+^ T cells), accelerated intraosseous tumor growth. SFB increased the activity of parathyroid hormone–related protein (PTHrP), a known driver of breast cancer bone metastasis produced by breast cancer cells. SFB promoted the PTHrP-induced expansion of intestinal Th17 cells and TNF^+^ T cells, their migration to bone marrow (BM) in tumor-bearing bones, and the BM expression of TNF, IL-17, and RANKL, cytokines that promote bone resorption. Demonstrating the functional relevance of IL-17 and TNF, silencing of either IL-17 or TNF blunted the growth of bone lesions in SFB^+^ mice. Thus, the abundance of SFB in the microbiome of mice and of SFB-like taxa in humans may determine the potency of PTHrP and the rate of growth of bone metastasis in breast cancer. Targeting the gut microbiota may represent a novel therapeutic strategy for breast cancer bone metastasis.

## Abstract 2

Gut bacteria called SFB speeds up breast cancer bone spread in mice through immune cells-mediated inflammation, indicating modulating gut microbiome could help patients

## Graphical abstract

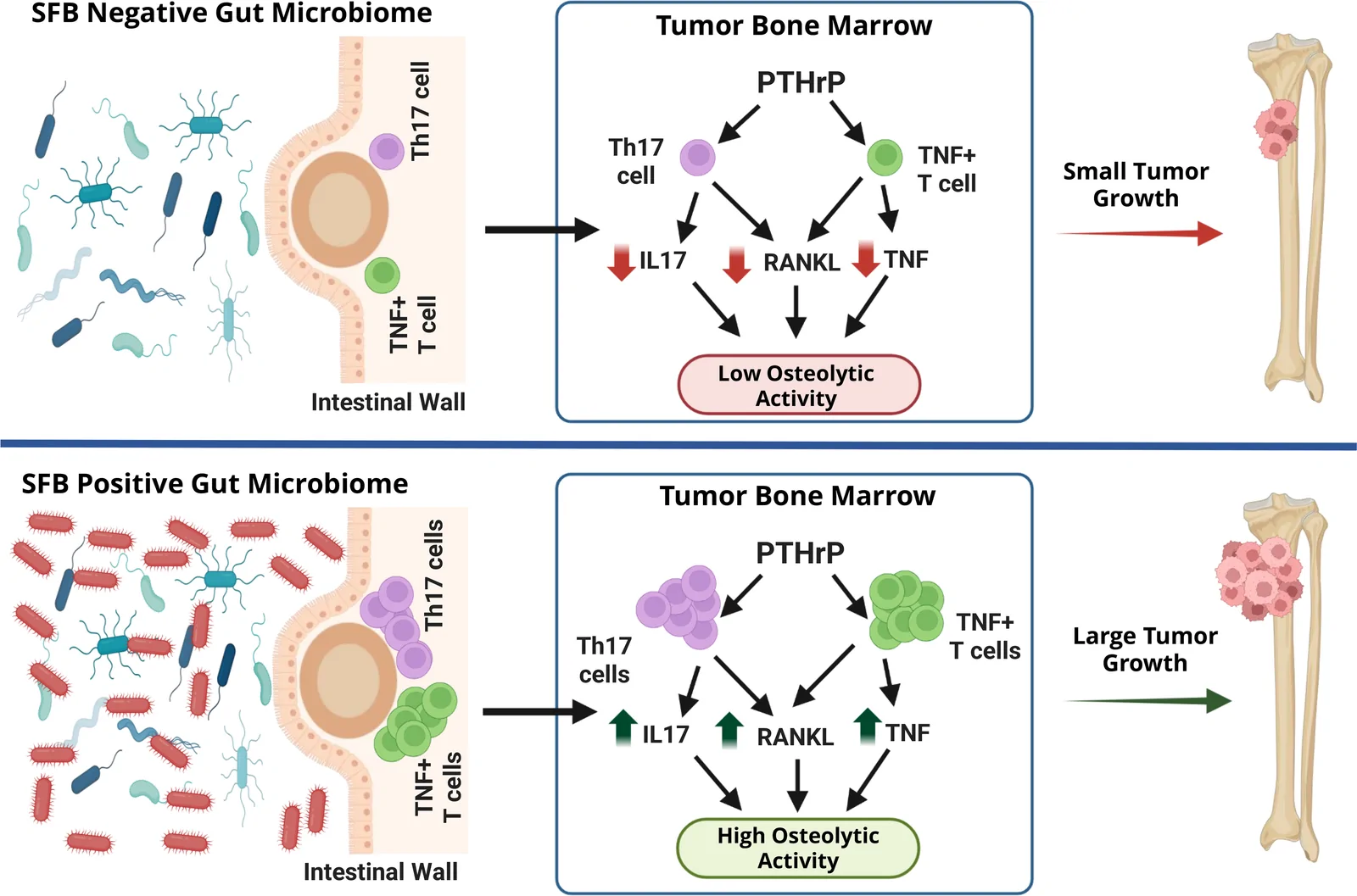

## Introduction

The gut microbiome is increasingly recognized as a critical regulator of bone in health and disease. Studies in humans and animal models have implicated the gut microbiome as a regulator of bone mineral density (1), postnatal skeletal development (2), bone tissue material properties (3), bone mineral absorption (4), and the pathogenesis of osteoporosis (5). In addition, the microbiome conditions the skeletal response to bone-regulating hormones including estrogen (6, 7), parathyroid hormone (PTH) (8–10), and glucocorticoids (11). We have shown that the gut microbiome contributes to the immunologic restraint of melanoma bone metastasis (12), while reports from other laboratories have implicated gut microbiome composition in cancer risk (13), cancer progression (14, 15), and response to immunotherapy (16, 17).

Studies in breast cancer have shown that dysbiosis enhances breast tumor cell dissemination to distant organs (18) and promotes inflammation within the mammary gland, enhancing the recruiting of pro-tumorigenic myeloid immune cells into the tumor microenvironment, thereby promoting cancer growth (18). Reports have also shown that the microbiome influences metastatic niches like the bone marrow (BM), where complex immune cell interactions can either suppress or promote secondary tumor growth (19, 20). This evidence highlights the microbiome’s potential as a target for diagnostic and therapeutic strategies aimed at preventing metastatic disease (21).

Breast cancer bone metastases are driven, in part, by parathyroid hormone–related protein (PTHrP), a factor produced by osteoblasts and breast cancer cells (22). PTHrP contributes to the development and growth of bone metastasis by regulating morphogenesis, cell proliferation, differentiation, apoptosis, and bone resorption (23). PTHrP shares limited sequence homology with PTH, a systemic hormone produced by the parathyroid glands. PTHrP binds to the same receptor as PTH, which is known as PTH/PTHrP receptor 1 (PTHR1) (24). This receptor is expressed by cells of the osteoblastic lineages (25) and by T cells (26). Ligand binding to PTHR1 induces production of critical osteoclastogenic cytokines including TNF-α (TNF), IL-17, and RANKL. Stimulation of osteoclastic bone resorption leads to release of TGF-β and other growth factors from the bone matrix, which further fuels tumor growth in a vicious cycle (27).

Studies in preclinical models and in humans (28) have established the contribution of the gut microbiome as a mediator of the effects of PTH in bone (8, 9, 29). Mechanistically, gut bacteria and bacterial products expand and activate intestinal TNF-producing T cells (TNF^+^ T cells) and Th17 cells that migrate to the BM in response to chemokine gradients induced by PTH. Once in the BM, these T cells release TNF and IL-17 (8). IL-17 stimulates osteocytic RANKL expression, a pivotal inducer of osteoclastogenesis and bone loss (30), while TNF potentiates the activity of RANKL (31). In mice, Th17 cells derive from naive intestinal CD4^+^ T cells primarily in response to segmented filamentous bacteria (SFB), which are spore-forming, Gram-positive commensal bacteria (32–34). An SFB-containing microbiome is also capable of promoting the expansion of TNF^+^ T cells (8). Accordingly, mice lacking SFB in their microbiome are protected against the bone-wasting activity of PTH, while those with SFB in their microbiome respond to PTH with an expansion of TNF^+^ T cells and Th17 cells and bone loss (8). Widespread human SFB species have recently been described (35), although the capacity of human SFB to induce Th17 cells remains to be determined. In addition, over 20 non-virulent gut bacteria are known to induce Th17 cells in humans (36, 37). Many of these species also induce TNF^+^ T cells (38, 39).

Since PTHrP and PTH have overlapping activities in bone and activate the same receptor, PTHrP-driven bone activity may also be regulated by SFB. Elucidating this gut-microbiota-bone axis in the context of PTHrP signaling could reveal novel therapeutic targets to mitigate bone metastatic progression in breast cancer patients. In the present study, we analyzed cell populations involved in the immune response to cancer, known to be regulated by the microbiome. We showed that SFB plays a critical role in promoting breast cancer bone metastatic growth in mice by enhancing the activity of PTHrP. SFB-induced activity of PTHrP was mediated by expansion and activation of intestinal TNF^+^ T cells and Th17 cells, their migration to the BM, and local release of TNF, IL-17, and RANKL. Thus, the abundance in the microbiome of SFB in mice and of SFB-like taxa in humans may determine the rate of growth of bone metastasis in breast cancer. Targeting the gut microbiota may represent a novel therapeutic strategy for breast cancer bone metastasis.

## Results

### The presence of SFB in the gut microbiome increases tumor growth in bone.

To investigate whether the gut microbiota influences the progression of bone metastasis, intratibial injections of luciferase-expressing PyMT breast cancer cells, a well-established mouse model of orthotopic and metastatic breast cancer (40, 41), were performed in 10-week-old C57BL/6 mice. To assess the role of SFB in bone cancer growth, we compared tumor growth in mice with SFB (SFB^+^) and without SFB (SFB^–^) in their gut microbiome. C57BL6/J SFB^–^ mice were purchased from The Jackson Laboratory (JAX). SFB^+^ JAX mice were generated by oral gavaging of SFB^–^ JAX mice with a liquid suspension of fecal pellets collected from SFB-monocolonized mice, as previously described (8, 12). In addition, C57BL6/TAC SFB^+^ mice were purchased from Taconic Biosciences (TAC). Stool bacterial DNA was analyzed by PCR to confirm the SFB status of these mice (Supplemental Figure 1, A and B; supplemental material available online with this article; https://doi.org/10.1172/jci.insight.207567DS1). In our first experiment, we analyzed bone tumor growth in SFB^–^ JAX mice and SFB^+^ TAC mice. In Vivo Imaging System (IVIS) imaging of tumor-bearing tibia on day 14 revealed that intratibial injection of PyMT cells induced a lower tumor burden in SFB^–^ JAX mice than in SFB^+^ TAC mice (Figure 1, A and B). Micro-CT scans were performed to measure tumor site total bone volume (BV), an index that reflects the trabecular and cortical bones covering the whole tumor site, and tibia trabecular BV, an index of the volume of the trabecular compartment. These measurements revealed a less severe loss of tumor site BV and trabecular BV in SFB^–^ JAX than in SFB^+^ TAC mice (Figure 1, C and D). To control for differences in genetic makeup and microbiota composition in tumor growth in mice of the same strain but differing in SFB status, we compared bone tumor growth in SFB^–^ and SFB^+^ JAX mice. We again found that intratibial injection of PyMT cells induced lower bone tumor growth and less severe loss of tumor site BV and trabecular BV in SFB^–^ mice than in SFB^+^ JAX mice (Figure 1, E–H).

To model colonization with SFB-like bacteria resulting from transmission from housemates or pets, and to further investigate its relevance for enhanced bone tumor growth, we cohoused SFB^–^ JAX mice with SFB^+^ TAC mice or SFB^–^ and SFB^+^ JAX mice in a 1:1 ratio for 2 weeks using established methods (2). Analysis of stool bacterial DNA confirmed that cohousing resulted in the conversion of SFB^–^ mice into SFB^+^ mice (Supplemental Figure 2, A and B). IVIS imaging of tumor-bearing mice and ex vivo micro-CT analysis on day 14 revealed that intratibial injection of PyMT cells induced a lower tumor burden and a smaller loss of BV in SFB^–^ mice compared with either SFB^+^ mice or cohoused animals (Figure 2, A–H). Together, these findings demonstrated that the presence of SFB in the gut microbiome potentiated PyMT-induced bone tumor growth.

### SFB induces the differentiation of intestinal Th17 cells and TNF^+^ T cells and their migration to the intratibial tumor site.

Since SFB is known to induce naive T cell differentiation into Th17 cells and TNF^+^ T cells (8), we analyzed the frequency and the mean fluorescence intensity (MFI) of IL-17 and TNF of these T cell populations in the Peyer’s patches (PPs) and tumor site BM of SFB^–^ and SFB^+^ JAX mice. MFI is a quantitative measure of the mean cytokine protein production per cell.

The gating strategy for identifying these T cell populations through flow cytometry is presented in Supplemental Figure 3, A and B. Because the measurement of the absolute number of PP cells is technically challenging due to variability of the size of the collected PP tissue, Th17 cells and TNF^+^ T cells in PPs were quantified as percentage of total cells. As expected, control no-tumor SFB^+^ mice had a higher relative frequency and MFI of PP Th17 cells and TNF^+^ T cells than SFB^–^ mice (Figure 3, A and B). In SFB^+^ mice, the injection of PyMT tumor cells did not significantly affect the relative frequencies and MFI of PP Th17 cells or TNF^+^ T cells, as no differences were observed between the tumor-free and tumor-bearing groups (Figure 3, A and B). However, injection of PyMT tumor cells increased the relative frequency, absolute number, and MFI of Th17 cells and TNF^+^ T cells in tumor site BM, and did so more potently in SFB^+^ than in SFB^–^ mice (Figure 3, C and D). Analysis of transcript levels of the key osteoclastogenic cytokines produced by Th17 cells and TNF^+^ T cells revealed higher *Il17*, *Tnf*, and *Rankl* mRNA levels in the tumor BM of SFB^+^ JAX mice than in the tumor BM of SFB^–^ JAX mice (Figure 3E). Measurements of RANKL protein by ELISA confirmed the presence of higher RANKL levels in the tumor BM of SFB^+^ JAX mice than in the tumor BM of SFB^–^ JAX mice (Figure 3F).

Similarly, these cytokines were higher in SFB^+^ TAC mice than in SFB^–^ JAX mice (Supplemental Figure 4). Together, these data suggest that the presence of SFB in the gut microbiome accelerates PyMT bone tumor growth by expanding Th17 cells and TNF^+^ T cells, and by increasing their production of osteoclastogenic cytokines at the tumor site.

Since Th17 cells possess context-dependent pro- and antitumor functions, we analyzed the phenotype of Th17 cells residing in tumor site BM. Subsets of Th17 cells expressing the pro-tumorigenic markers FoxP3 and IL-10 (regulatory Th17 cells) and those expressing the anti-tumorigenic markers IFN-γ and T-bet (Th1-like Th17 cells) were quantified by flow cytometry in SFB^+^ TAC mice and SFB^–^ JAX mice. The gating strategy for identifying these T cell populations is shown in Supplemental Figure 5. Pro- and anti-tumorigenic Th17 cells were present in the BM at tumor sites in both SFB^+^ and SFB^–^ mice. However, the frequency of pro-tumorigenic Th17 cells was higher than the frequency of anti-tumorigenic Th17 cells in both SFB^+^ and SFB^–^ mice (Supplemental Figure 6, A–D).

The intestine functions as a reservoir of Th17 cells and TNF^+^ T cells. From the intestine, these cells migrate to distant sites of inflammation driven by chemokine gradients (7, 12, 42–44). Thus, the expansion of T cells in the tumor site BM may result from the egress of intestinal T cells and their homing to the BM. To directly investigate whether tumor growth induced the migration of intestinal Th17 cells and TNF^+^ T cells to the tumor site, we utilized C57BL/6 SFB^–^ and SFB^+^ Kaede mice using methods established in our laboratory (7, 12, 45). Kaede mice enable sensitive tracking of leukocyte migration using the photoconvertible protein Kaede, which shifts fluorescence from green (518 nm) to red (582 nm) upon UV exposure (350–410 nm). Photoconverted (KaedeR) cells can be detected in other organs by flow cytometry without affecting cellular function or T cell homing (46). Intratibial injections of control (CT) PyMT cells were performed in 10-week-old SFB^–^ and SFB^+^ Kaede mice. SFB^+^ Kaede mice were generated by gavaging of SFB^–^ Kaede mice with a liquid suspension of fecal pellets collected from SFB-monocolonized mice, as previously described (8). Stool DNA was analyzed to confirm the presence of SFB in Kaede mice monocolonized with SFB (Supplemental Figure 7). Nine days after the injection of PyMT cells, a time point when bone lesions were established and rapidly growing, all animals were subjected to surgical laparotomy, and 4 PPs per mouse were photoactivated by exposure to a 390 nm light for 2 minutes. To ensure that no cells outside the intestine were photoconverted, the whole body was covered with an aluminum foil blanket. Mice were sacrificed 24 hours after photoconversion, and the number of KaedeR Th17 cells and TNF^+^ T cells was measured by flow cytometry in PPs and tumor site BM.

Confirming that injection of PyMT breast cancer cells induces tumor growth in Kaede mice, and that tumor growth was SFB dependent, IVIS imaging of the tumor-bearing mice revealed significant tumor burden in SFB^+^ Kaede mice and lower tumor growth in SFB^–^ Kaede mice (Figure 4, A and B). Control healthy SFB^+^ mice had a higher frequency of PP KaedeR Th17 and KaedeR TNF^+^ T cells compared with tumor-injected mice (Figure 4, C and D). By contrast, no-tumor and tumor-injected SFB^–^ mice had similar numbers of PP KaedeR Th17 and KaedeR TNF^+^ T cells (Figure 4, C and D). These findings suggest that the egress of Th17 cells and TNF^+^ T cells increased during bone cancer growth, and that the presence of SFB in the gut microbiome resulted in higher PP egress of Th17 cells and TNF^+^ T cells. Analysis of the BM revealed that in SFB^+^ mice the absolute and relative frequencies of KaedeR Th17 and KaedeR TNF^+^ T cells were significantly higher in the BM at the tumor site than in the BM of the contralateral healthy tibia or the BM of no-tumor mice (Figure 4, E and F). These findings indicate that intestinal Th17 cells and TNF^+^ T cells specifically home to tumor BM. Consistent with lower tumor growth, in SFB^–^ mice the frequency of KaedeR Th17 and KaedeR TNF^+^ T cells in tumor BM was higher than that in contralateral BM, but markedly lower than that in the corresponding tumor group of SFB^+^ mice (Figure 4, E and F). SFB^+^ mice had a higher number of KaedeR TNF^+^ T cells but not of KaedeR Th17 cells, as compared with SFB^–^ mice. Since the homing of TNF^+^ T cells to the BM is required to upregulate CCL20, (8), the chemokine ligand responsible for the BM homing of Th17 cells, these findings suggest that the homing of TNF^+^ T cells to the contralateral BM induced by SFB is not sufficient to upregulate the levels of CCL20 to the amount required for attracting Th17 cells.

### SFB does not regulate tumor cell–intrinsic proliferation, survival, or immune evasion via metabolite production.

In addition to regulating tumor growth by promoting intestinal T cell differentiation and activation, SFB could produce metabolites that regulate tumor cell proliferation, survival, or immune evasion. Regulatory metabolites produced by SFB must diffuse in the bloodstream to reach tumor cells. Therefore, we conducted a screening experiment in which serum from SFB^+^ or SFB^–^ mice was added to PyMT cell culture for 24–72 hours. PyMT cell proliferation and survival were assessed by flow cytometric measurement of annexin V. Immune evasion properties were assessed by measurement of the PyMT cell expression of MHC-I, PD-L1, CD80, CD47, and CD73 by flow cytometry. MHC-I^+^PD-L1^+^ cells were regarded as expressing an adaptive immune resistance phenotype (47, 48), and PD-L1^+^CD80^+^ PyMT cells were regarded as having a checkpoint-costimulatory rheostat phenotype (49, 50), while CD47^+^CD73^+^PD-L1^+^ PyMT cells were regarded as possessing an immune-evasive phenotype (51).

These experiments revealed that PyMT cells cultured for 24–72 hours in the presence of 0%–20% serum from SFB^+^ or SFB^–^ mice had similar proliferation, survival (Supplemental Figure 8), and immune evasion properties (Supplemental Figures 9 and 10).

The main metabolites produced by SFB are serum amyloid A (SAA) proteins. ELISA measurements confirmed the presence of higher SAA serum levels in SFB^+^ TAC mice than in SFB^–^ JAX mice (Supplemental Figure 11A). To assess whether SAA proteins contribute to the regulatory role of SFB in tumor cell activity, PyMT cells were cultured for 24–72 hours in the presence of SAA1. We found that physiologic concentrations of SAA1 failed to regulate PyMT cell proliferation, survival (Supplemental Figure 11B), or immune evasion properties (Supplemental Figure 12). However, supraphysiologic levels of SAA1 slightly decreased cell survival at 72 hours (Supplemental Figure 11B). Together these findings strongly suggest that SFB does not regulate PyMT cell activity through metabolites.

### Deletion of PTHrP in PyMT breast cancer cells reduces tumor burden and bone loss by diminishing the expansion of Th17 and TNF^+^ T cells at the tumor site.

To confirm the relevance of PTHrP for the growth of bone tumor lesions induced by PyMT cells, we generated PTHrP-KO PyMT cells. Successful knockout of PTHrP expression was confirmed through quantitative reverse transcription PCR (qRT-PCR) analysis of PTHrP transcripts in PyMT cells and ELISA measurement of the protein in cell culture–conditioned medium (Supplemental Figure 13, A and B). Control (CT) PyMT cells and PTHrP-KO PyMT cells were injected intratibially into SFB^–^ and SFB^+^ JAX mice. On day 14 after injection, SFB^–^ JAX mice injected with CT or PTHrP-KO PyMT cells had similar tumor burden and BV (Figure 5, A–D). By contrast, SFB^+^ mice injected with PTHrP-KO PyMT cells had an approximately 3.5-fold lower tumor burden and higher total and trabecular BV than those injected with CT PyMT cells (Figure 5, E–H). Consistent both with the capacity of PyMT cells to produce PTHrP and with the larger tumor growth in SFB^+^ mice than in SFB^–^ mice, assessment of PTHrP expression in tumor tissues derived from the bone lesions revealed higher levels of PTHrP expression in tumor-injected SFB^+^ mice as compared with SFB^–^ mice, and lower transcript and protein levels of PTHrP in mice injected with PTHrP-KO PyMT cells (Supplemental Figure 13, C and D).

In SFB^–^ mice, injection of CT or PTHrP-KO PyMT cells did not alter the relative frequency of PP Th17 cells (Figure 6A) and the relative frequency and the absolute number of BM Th17 cells (Figure 6B). To investigate the effects of PTHrP on the secretory activity of Th17 cells, we measured the MFI of IL-17. We found that injection of CT or PTHrP-KO PyMT cells did not affect BM Th17 cell MFI (Figure 6C). Injection of CT or PTHrP-KO PyMT cells in SFB^–^ mice did not affect the relative frequency of PP TNF^+^ T cells (Figure 6D), but it did equally increase the relative frequency and the absolute number of BM TNF^+^ T cells (Figure 6E). Injection of CT but not PTHrP-KO PyMT cells increased BM TNF^+^ T cell MFI (Figure 6F).

In SFB^+^ mice, neither injection of CT PyMT cells nor injection of PTHrP-KO PyMT cells altered the relative frequency of PP Th17 cells (Figure 6G). However, deletion of PTHrP blunted the tumor-induced increase in the relative frequency and MFI of BM Th17 cells (Figure 6, H and I). Similarly, injection of either CT or PTHrP-KO PyMT cells did not change the relative frequency of PP TNF^+^ T cells (Figure 6J). However, mice injected with CT PyMT cells exhibited a higher increase in the relative frequency and MFI of BM TNF^+^ T cells compared with those injected with PTHrP-KO PyMT cells (Figure 6, K and L). In SFB^+^ mice, CT PyMT cell but not PTHrP-KO PyMT cell injection increased *Il17*, *Tnf*, and *Rankl* expression at tumor sites (Supplemental Figure 14). Together, these data suggest that in the presence of SFB, PTHrP accelerates PyMT bone tumor growth by increasing the production of TNF, IL-17, and RANKL at the tumor site.

### Global deletion of IL-17 and TNF prevents the expansion and migration of Th17 and TNF^+^ T cells to the tumor site, leading to reduced tumor growth and bone loss.

To directly assess the functional significance of IL-17 and TNF in SFB-mediated tumor growth, CT PyMT cells were injected in 10-week-old SFB^+^ IL-17^–/–^ mice, TNF^–/–^ mice, and WT littermates (7, 8). IVIS imaging of the tumor-bearing mice on day 14 after injection revealed a lower tumor burden in IL-17^–/–^ mice compared with WT controls (Figure 7, A and B). Micro-CT scanning of tumor-bearing tibia revealed higher total and trabecular BV in IL-17^–/–^ mice compared with WT mice (Figure 7, C and D). As expected, PP and BM Th17 cells were undetectable in IL-17^–/–^ mice (Figure 7, E and F). WT and IL-17^–/–^ mice had similar frequencies of PP and BM TNF^+^ T cells, in both the tumor-free and tumor-injected groups (Figure 7, G and H). Analysis of cytokine transcripts revealed that silencing of *Il17* decreased *Rankl* expression in the BM of tumor-bearing mice, although it did not affect *Tnf* transcript levels. As expected, *Il17* transcript was detectable in WT littermates only (Supplemental Figure 15A). Experiments comparing TNF^–/–^ mice with WT littermates revealed a lower tumor burden in TNF^–/–^ mice than in WT mice (Figure 8, A and B) and a higher trabecular BV and tumor site total BV in TNF^–/–^ mice than in WT mice (Figure 8, C and D), demonstrating that silencing of TNF lowers tumor burden and bone waste. While silencing of *Tnf* did not alter the frequency of PP Th17 cells (Figure 8E), it did lower the number of Th17 cells in the tumor BM (Figure 8F), demonstrating a requirement for TNF for the migration of Th17 cells from the gut to the bone tumor lesions. As expected, TNF^+^ T cells were undetectable in the PPs and BM of TNF^–/–^ mice (Figure 8, G and H). Analysis of cytokine transcripts revealed that silencing of *Tnf* decreased *Il17* and *Rankl* expression in the BM of tumor-bearing mice. *Tnf* transcripts were not detectable in TNF^–/–^ mice (Supplemental Figure 15B).

## Discussion

We report that SFB potentiated the capacity of PTHrP to promote the growth of bone lesions in an animal model of breast cancer bone metastasis. SFB increased PTHrP-driven expansion of intestinal TNF^+^ T cells and Th17 cells, their migration to the BM of tumor-bearing bones, and the release of the osteoclastogenic cytokines TNF, IL-17, and RANKL. To our knowledge, this is the first report indicating a role for SFB, and thus the composition of the gut microbiome, in the tumor-promoting activity of PTHrP.

The relevance of SFB to the development of bone lesions was unveiled by comparison of the effects of PyMT cell injection in 3 mouse populations differing in SFB status, and confirmed by cohousing of SFB^–^ mice and SFB^+^ mice. Because mice are coprophagic, cohousing experiments provide a robust model to establish the efficiency of microbiome transmission between animals, and the detection of microbiome-induced phenotypes (52, 53).

We found that SFB^+^ mice exhibited higher frequencies of Th17 cells and TNF^+^ T cells in PPs and BM compared with SFB^–^ mice. The SFB-induced expansion of these intestinal T cells was similar in control and PyMT cell–injected mice, indicating that bone tumor growth did not regulate the size of the intestinal pool of Th17 cells and TNF^+^ T cells. By contrast, bone tumor–loaded SFB^+^ mice had higher frequencies of BM Th17 cells and TNF^+^ T cells compared with healthy control SFB^+^ mice. To determine whether the tumor-induced increase in the frequency of BM T cells was due to direct expansion of BM T cells or homing to the BM of intestinal T cells, we made use of Kaede mice (54). This strain has been successfully used to track the migration of intestinal immune cells to the BM (7, 12), kidney (44), mesenteric lymph nodes (55), and brain (56). Our experiments revealed that bone tumor growth specifically increased the tropism of Th17 cells and TNF^+^ T cells for the BM of tumor-bearing bones, and that the homing of intestinal T cells to the BM was potentiated by SFB. Further studies with Kaede mice remain to be conducted to determine whether PTHrP regulates egress of intestinal T cells or their homing to the BM. We did not find evidence that bone cells or bone-produced factors are required for intestinal Th17 cells and TNF^+^ T cells to home to the BM of tumor-bearing bones, but a possible contribution of bone-specific factors remains to be conclusively determined. These findings provide novel mechanistic insights into the role of microbiome species capable of inducing Th17 cells and TNF^+^ T cells in the development of bone lesions in breast cancer.

SFB and the whole-gut microbiota are likely to regulate the activity of PTHrP and the immune response to breast cancer not only by directly modulating T cell activation and trafficking, but also via the production of microbial metabolites. The main metabolites induced by SFB are serum amyloid A (SAA) proteins (33). SAA proteins are critical for the differentiation of intestinal Th17 cells and thus for PTHrP activity and tumor growth (57).

Additional metabolites that may play a regulatory role include short-chain fatty acids produced by commensal gut bacteria, which enhance antitumor T cell activity (58, 59), and gut-derived lipopolysaccharide, which accelerates tumor progression through PD-L1 upregulation on cancer cells, thus conferring resistance to T cell–mediated cytotoxicity (60). It should also be noted that the intestinal immune shifts induced by SFB cause minor microbiome composition changes, primarily by altering the mucosal environment and the secretion of antimicrobial peptide (33). Thus, the effects of SFB on tumor growth might, in part, be mediated by SFB-induced changes in microbiome composition.

We did not investigate the mechanism responsible for the homing of intestinal Th17 cells and TNF^+^ T cells to the BM, because in previous reports we have shown that the migration of these populations is driven by chemokine gradients. Specifically, TNF^+^ T cells and Th17 cells egress the intestine through an S1PR1-dependent mechanism, while the influx of TNF^+^ T cells and Th17 cells into the BM is guided by CXCR3 and its ligand CXCL9, and by CCR6 and its receptor CCL20 (7, 8, 61). We reported that PTH regulates the migration of T cells via a chemokine-mediated mechanism activated by TNF (8). Further studies will be necessary to investigate whether PTHrP regulates T cell migration with the same mechanisms.

The role of TNF^+^ T cells and Th17 cells in cancer has been investigated previously (62–65). Studies have shown that Th17 cells exhibit remarkable functional plasticity, enabling them to adopt either immunosuppressive or effector phenotypes depending on tumor microenvironmental cues. For example, in TGF-β–rich immunosuppressive environments, Th17 cells can convert into regulatory-like cells that promote angiogenesis and recruit myeloid-derived suppressor cells, thereby supporting tumor progression (62). Conversely, exposure to IL-12 or IL-23 can redirect Th17 cells to transdifferentiate into Th1-like effector cells, producing IFN-γ and activating cytotoxic CD8^+^ T cells to mediate tumor suppression (63–65).

IL-17 and TNF promote osteoclastogenesis both directly and by inducing RANKL expression by osteoblastic cells (66, 67). Reflecting the size of the pool of Th17 cells and TNF^+^ T cells, expression of IL-17, TNF, and RANKL proteins in the tumor site BM was higher in SFB^+^ mice than in SFB^–^ mice. In this study, the functional role of TNF and IL-17 was demonstrated by the finding that the global deletion of either IL-17 or TNF blunted bone tumor growth in SFB^+^ mice. We found that deletion of TNF prevented the tumor-induced expansion of BM Th17 cells, whereas deletion of IL-17 did not blunt the frequency of BM TNF^+^ T cells. These findings confirmed a requirement for TNF for Th17 expansion. Interestingly, deletion of IL-17 blunted the tumor-induced increase in the BM expression of RANKL but not of TNF, indicating that IL-17 and RANKL are more relevant than TNF in SFB-dependent bone tumor growth. Since osteoclastic bone resorption is pivotal for bone lesion progression (68), these findings provide a mechanism to link the presence of SFB in the gut microbiome with faster bone tumor lesion growth.

Our findings are consistent with prior reports showing that IL-17 activates oncogenic pathways to enhance tumor cell survival (64, 69). However, it should be noted that under specific immunotherapeutic conditions, IL-17–producing cells can enhance antitumor immunity, illustrating the double-edged nature of this cytokine axis (64, 69). Similarly, TNF exhibits a dual role that depends on signaling via TNFR1 or TNFR2 and the chronicity of exposure. Acute TNF can induce tumor cell apoptosis and enhance T cell priming, whereas chronic TNF in the tumor microenvironment activates NF-κB–dependent survival pathways, stabilizes regulatory T cells, induces PD-L1 expression, and sustains IL-17–driven inflammatory circuits that promote tumor growth and myeloid cell recruitment (70–74). Collectively, these studies highlight that the dichotomous roles of IL-17 and TNF in cancer are dictated by microenvironmental cues, cytokine context, and T cell plasticity, making them both potential targets and modulators in cancer therapy.

Our main experimental model was the intratibial injection of PyMT cells. Although this method bypasses key steps of the metastatic process, this approach delivers cells directly into the BM cavity, enabling controlled and reproducible establishment of localized bone tumors, and assessment of tumor-bone and immune-bone interactions independent of earlier steps in the metastatic cascade (75, 76). Our findings were not validated in an intracardiac PyMT cell injection model because in preliminary studies this experimental modality resulted in the development of lethal intrapulmonary lesions before the establishment of bone lesions.

In summary, this study reveals an unexpected extraskeletal factor, namely the gut microbiome, as a determinant of the activity of PTHrP and highlights the relevance of Th17 cell– and TNF^+^ T cell–inducing taxa in the development of osteolytic bone lesions in a mouse model of breast cancer. Our findings have significant clinical implications because the presence or absence of the human equivalent of a Th17 cell–inducing microbiota in patients affected by breast cancer may determine the clinical manifestation of this condition. Thus, stool microbiome sequencing might offer a potential biomarker for bone metastatic risk, while modification of the gut microbiome composition with pre/pro- or postbiotic interventions might provide a novel therapeutic strategy against bone metastasis in breast cancer.

## Methods

### Sex as a biological variable.

Female mice were used in our study, because breast cancer predominantly affects women.

### Mice.

C57BL/6J mice were acquired from both The Jackson Laboratory (JAX) and Taconic Biosciences (TAC). The microbiome of TAC mice harbors SFB (SFB^+^), whereas JAX mice are without SFB (SFB^–^). SFB^–^ Kaede mice [B6.Cg-c/c Tg(CAGtdKaede)15Utr] were obtained from RIKEN BioResource Research Center. SFB^–^ TNF^–/–^ mice (B6.129S6-Tnf<tm1Gkl>/J) and SFB^–^ IL-17^–/–^ mice (B6.Cg-Il17a/Il17ftm1.1Impr Thy1a/J) were purchased from JAX. All mice were shipped to the same vivarium room at Emory University and housed under specific pathogen–free conditions. They received γ-irradiated 5V5R chow (Purina Mills) and autoclaved water ad libitum. The facility maintained a temperature of 23°C (± 1°C), 50% humidity, and a 12-hour light/12-hour dark cycle. Mice were acclimatized for at least 3 days prior to experiments.

### Generation of SFB-colonized mice.

To generate SFB^+^ JAX and Kaede mice, liquid suspensions of fecal pellets from SFB-mono-associated mice were orally gavaged to SFB^–^ JAX and Kaede mice as described by previous studies (8, 12). SFB status was verified by qPCR using SFB-specific 16S rDNA gene primers 5′-GACGCTGAGGCATGAGAGCAT-3′, forward, and 5′-GACGGCACGGATTGTTATTCA-3′, reverse (8, 12).

### Cohousing.

For the cohousing experiment, 8-week-old female SFB^–^ JAX and SFB^+^ TAC mice or SFB^–^ JAX and SFB^+^ JAX mice (1:1 ratio) were cohoused for 2 weeks. All mice were supplied with sterile water and chow. Control groups were housed separately. Since mice are coprophagic, cohousing facilitated the equalization of the gut microbiome (2).

### PyMT-BO1-GFP-Luc cell line culture and CRISPR/Cas9–mediated gene knockout of PTHrP gene (Pthlh).

The PyMT-BO1-GFP-Luc cell line (PyMT) was a gift from Roberta Faccio, Washington University School of Medicine (St. Louis, Missouri, USA). PyMT cells were cultured in DMEM with 10% FBS in a humidified chamber under standard culture conditions. Luciferase activity was checked before proceeding with in vivo experiments. For PTHrP gene (*Pthlh*) knockout, the CRISPR/Cas9 system from Santa Cruz Biotechnology (sc-422491) was used according to the manufacturer’s instructions. Briefly, cells were seeded in a 6-well plate and cotransfected at 60%–70% confluence using UltraCruz Transfection Reagent (sc-395739, Santa Cruz Biotechnology) with 1.5 μg of the target-specific CRISPR/Cas9 KO Plasmid and 1.5 μg of the corresponding HDR Plasmid. A control group was transfected with a non-targeting control CRISPR/Cas9 Plasmid (sc-418922). Forty-eight hours after transfection, successfully transfected cells were selected with an optimal concentration of puromycin for 7 days to establish a stable polyclonal population. PTHrP knockout generated in the PyMT cells was designated as PTHrP KO, while cells transfected with a non-targeting control CRISPR/Cas9 vector served as the wild-type control (PTHrP CT).

The efficacy of the knockout was validated at the transcriptional and translational levels. Total RNA was extracted from PyMT cells and tumor tissue from the animal study using TRIzol Reagent (Thermo Fisher Scientific), reverse-transcribed with the SuperScript III First Strand kit (Invitrogen), and subjected to qRT-PCR using a TaqMan assay with a PTHrP-specific probe (Mm00436057_m1, Thermo Fisher Scientific). Transcript levels were normalized to 18s rRNA (Mm03928990_g1, Thermo Fisher Scientific) and calculated using the 2^–ΔΔCt^ method. Concurrently, PTHrP protein secretion was quantified from cell culture supernatants using a Mouse Parathyroid Hormone Related Protein (PTHrP) BioAssay ELISA Kit (catalog 517531, USBiological Life Sciences), following the manufacturer’s protocol, and only those colonies of cell populations that showed no PTHrP protein levels in conditioned medium were maintained and used for in vivo experiments. For subsequent in vivo experiments, PTHrP levels in tumor tissue homogenates were also measured by ELISA. Briefly, 5 mg of tumor tissue was rinsed, homogenized in 0.1% SDS lysis buffer with PMSF, and subjected to freeze-thaw cycle to release intracellular PTHrP. Centrifugation was performed at 1,500 × *g* to eliminate the cell lysate debris, and 50 μL of the supernatant was used to perform ELISA per manufacturer protocol. This dual approach confirmed the complete ablation of PTHrP expression in the PTHrP-KO cells at both the mRNA and protein levels.

### Intratibial PyMT cell injection.

Intratibial injections were performed by administration of 1 × 10^5^ luciferase-expressing PyMT cells (in 10 μL PBS) into 10-week-old female mice (12). Mice were monitored daily and euthanized after 14 days. Control groups comprised PBS-injected contralateral tibiae and PPs from no-tumor cohorts.

### IVIS-based analysis of tumor growth.

Tumor growth analysis was achieved using the PerkinElmer IVIS Spectrum In Vivo Imaging System. Briefly, mice were injected s.c. with 150 mg/kg d-luciferin potassium salt (122799, PerkinElmer), anesthetized using isoflurane, and imaged 15 minutes after injection as previously described (12). On day 14, imaging was conducted to assess intratibial tumor growth. For analysis, total photon flux (photons per second) was measured from a fixed region of interest in the tumor area using Living Image 4.50 software.

### Micro-CT–based analysis of tibia trabecular bone volume and tumor site total bone volume.

Tumor-bearing tibia and the contralateral control tibia bones were imaged in the axial plane at 70 peak kV, 114 μA, 500 projections per rotation, integration time of 300 milliseconds, then reconstructed using the 1,200 mg hydroxyapatite/cm^3^ beam hardening correction to generate axial images with 12 μm isotropic voxels by using a Scanco micro-CT 40 scanner (Scanco Medical). The volume of interest (VOI) for the tibia trabecular bone volume (BV) analysis was 70 slices from the central tip of the growth plate, covering the trabecular metaphysis region. The VOI for the tumor site total BV analysis was 500 slices from the central tip of the growth plate, covering the total tumor-bearing region of the tibia (12). 3D models were generated with the tumor site total BV VOI for the representation.

### PP and BM single-cell suspension.

Small intestine tissue was harvested and cleared of fecal contents for PP isolation. Identified PPs were excised in ice-cold RPMI 1640 medium (1 mL), mechanically dissociated using the plunger of a 2.5 mL syringe, and gently passaged through a cell strainer placed over a 50 mL tube. BM cells were isolated from tumor-bearing tibia via PBS flushing. BM samples underwent RBC lysis to remove RBCs from the samples. Single-cell suspensions of PPs and BM were subsequently prepared and analyzed via flow cytometry using established protocols (8, 12).

### Kaede mouse cell photoconversion and cell trafficking analysis.

Kaede mice express a photoconvertible protein that, when exposed to near-UV light (350–410 nm), shifts fluorescence from green (518 nm) to red (582 nm). Experiments with Kaede mice were performed as previously described (7, 12, 45). Ten-week-old SFB^+^ and SFB^–^ Kaede mice were subjected to intratibial PyMT tumor cell injection. A laparotomy was performed 9 days later, the cecum and distal small intestine were eviscerated, and the 4 most proximal PPs were located and illuminated with 390-nm-wavelength light for 2 minutes each. Aluminum foil was used to protect tissue other than target PPs from light during exposure. Cell suspensions from PPs and BM were prepared 24 hours after photoconversion. A single-cell suspension was prepared for flow cytometry analysis to enumerate red fluorescent TNF^+^ T cells and Th17 cells.

### Flow cytometry.

Flow cytometry was performed on a Cytek Aurora FACS or BD FACSymphony system, with subsequent data analysis conducted in FlowJo software (Tree Star). To detect surface markers, cells were stained with purified anti–mouse CD16/32 (Fc-blocking antibody, clone 93, catalog 101302), BV510-CD45 (clone 30-F11, catalog 103138), BV421-TCRβ (clone H57-597, catalog 109230), AF700-CD3 (clone 17A2, catalog 100216), PerCP/Cy5.5-CD4 (clone RM4-5, catalog 100540), and BV711-CD8 (clone 53-6.7, catalog 100748) (all from BioLegend). Viable cells were identified using either the Zombie NIR Fixable Viability Kit (catalog 77184, BioLegend) or the LIVE/DEAD Fixable Yellow Dead Cell Stain Kit (catalog L34959, Thermo Fisher Scientific). For intracellular staining, cells were stimulated with an activation cocktail containing monensin (BioLegend) at 37°C for 12 hours. Anti-mouse PE– or APC–IL-17A (clone eBio17B7, catalog 12-7177-81 or 17-7177-81) (Thermo Fisher Scientific) and APC-TNF (clone MP6-XT22, catalog 554420) (BD Biosciences) were added after cell fixation and permeabilization with Intracellular Fixation & Permeabilization Buffer Set (Thermo Fisher Scientific). For the analysis of Th17 cell subtypes, separate panels for Treg-like Th17 and Th1-like Th17 identification were designed. Viable cells were detected with either Zombie NIR Fixable Viability Kit (catalog 77184, BioLegend) or Zombie Fixable Yellow Dead Cell Stain Kit (catalog 77168, BioLegend). Surface markers were stained with purified anti–mouse CD16/32 (Fc-blocking antibody, clone 93, catalog 101302), PerCP-CD45 (clone 30-F11, catalog 103129), APC/Cy7-CD45 (clone S18009F, catalog 157204), AF700-CD3 (clone 17A2, catalog 100216), PE/Cy7-CD4 (clone GK1.5, catalog 100422), and PerCP/Cy5.5-CD4 (clone RM4-5, catalog 100540) (all from BioLegend). Intracellular staining was performed with anti-mouse APC–IL-17A (clone TC11-18H10.1, catalog 506916), AF488–IL-10 (clone JES5-16E3, catalog 505013), BV421-FOXP3 (clone MF-14, catalog 126419), PE/Cy7–T-bet (clone 4B10, catalog 644824), and BV421–IFN-γ (clone XMG1.2, catalog 505829) (all from BioLegend) and PE-RORγt (clone Q31-378, catalog 562607, BD Biosciences). For the analysis of immune evasion phenotypes of PyMT breast cancer cells, cells were stained with FITC–MHC-I H-2Kb/H-2Db (clone 28-8-6, catalog 114606), PE/Cy7–PD-L1 (clone 10F.9G2, catalog 124314), APC-CD80 (clone 16-10A1, catalog 104714), PE/Dazzle594-CD47 (clone miap301, catalog 127521), and PerCP/Cy5.5-CD73 (clone TY/11.8, catalog 127213) (all from BioLegend).

The cell viability assay was performed using Annexin V–FITC/PI reagent (catalog HY-K1073, MedChemExpress) per the manufacturer’s protocol to analyze the effect of SFB^–^ JAX and SFB^+^ mouse serum and SAA1 (catalog HY-P79122, MedChemExpress) on PyMT cancer cells.

### ELISA.

Serum SAA1/2 levels were measured using an ELISA kit obtained from R&D Systems (catalog MSAA00). The assay uses antibodies raised against recombinant mouse SAA1 (which shares about 91% sequence identity with SAA2) and displays cross-reactivity to accurately quantitate the natural forms of both SAA1 and SAA2. RANKL levels in BM cell culture supernatants were quantified with an ELISA kit by RayBiotech (catalog ELM-TRANCE). ELISA assays were performed following the manufacturer’s instructions, including sample preparation, addition of standards and samples, incubation, washing, and detection steps. The concentrations of SAA1/2 and RANKL were calculated from standard curves generated using the standards provided with the respective kits.

### qRT-PCR and primers.

Total RNA was extracted with TRIzol Reagent (Thermo Fisher Scientific) per the manufacturer’s instructions. cDNA was synthesized from mRNA using the SuperScript III First Strand kit (Invitrogen). qRT-PCR was performed to measure *Rankl*, *Il17*, and *Tnf* expression levels in tumor-bearing BM cells. Changes in relative gene expression between groups were calculated by the 2^–ΔΔCt^ method with normalization by *Actin* mRNA transcript level. Primer sequences used for genes were: *Rankl*, 5′-CCTGATGAAAGGAGGGAGCA-3′ (forward) and 5′-TGGAATTCAGAATTGCCCGA-3′ (reverse); *Il17*, 5′-TGACGCCCACCTACAACATC-3′ (forward) and 5′-CATCATGCAGTTCCGTCAGC-3′ (reverse); *Tnf*, 5′-AACTCCAGGCGGTGCCTAT-3′ (forward) and 5′-TGCCACAAGCAGGAATGAGA-3′ (reverse); and *Actin*, 5′-GTTGTCGACGACGAGCG-3′ (forward) and 5′-GCACAGAGCCTCGCCTT-3′ (reverse).

### Statistics.

All data were expressed as mean ± SD. All data were normally distributed according to the Shapiro-Wilk normality test. Tumor volume, measured by luminescence, was log_10_-transformed to ensure normality. Data were compared for each experiment separately. All data were analyzed by unpaired 2-tailed *t* test, 1-way ANOVA, or 2-way ANOVA, as appropriate, applying the Bonferroni correction for multiple comparisons. This analysis included the main effects for tumor versus no tumor and SFB or PTHrP, IL-17, or TNF deletion, plus the statistical interaction between these variables. When the statistical interaction was statistically significant (*P* < 0.05) or suggestive of an important interaction, then unpaired 2-tailed *t* tests were used to compare the differences between the treatment means for each animal strain, applying Bonferroni’s correction for multiple comparisons. *P* values less than 0.05 were considered statistically significant.

### Study approval.

The Institutional Animal Care and Use Committee of Emory University approved all animal procedures, ensuring compliance with federal regulations for the protection of research animals.

### Data availability.

Supporting data values underlying the figures are provided in the Supporting Data Values XLS file.

## Author contributions

RP designed the studies. NKR, S Pal, and HYD performed the research. S Pal, S Patil, and NKR analyzed the data. RP, MNW, NKR, and HYD wrote or edited the manuscript. NKR and S Pal contributed equally to this work. The order of co–first authorship was determined by mutual agreement between the two authors and based on the relative scope of contribution, data acquisition, and manuscript preparation.

### AI disclosure

The authors declare that no AI tools were used in the original research for or writing of this article.

## Conflict of interest

The authors have declared that no conflict of interest exists.

## Funding support

This work is the result of NIH funding, in whole or in part, and is subject to the NIH Public Access Policy. Through acceptance of this federal funding, the NIH has been given a right to make the work publicly available in PubMed Central.

- National Institutes of Health grants RR028009 to RP, and AG062334, AR068157, AR070091, and AR79298 to MNW.
- Biomedical Laboratory Research and Development Service of the VA Office of Research and Development (grant 5I01BX000105) to MNW.

## Supplementary Material

Supplemental data

Supporting data values

## Figures and Tables

**Figure 1:**
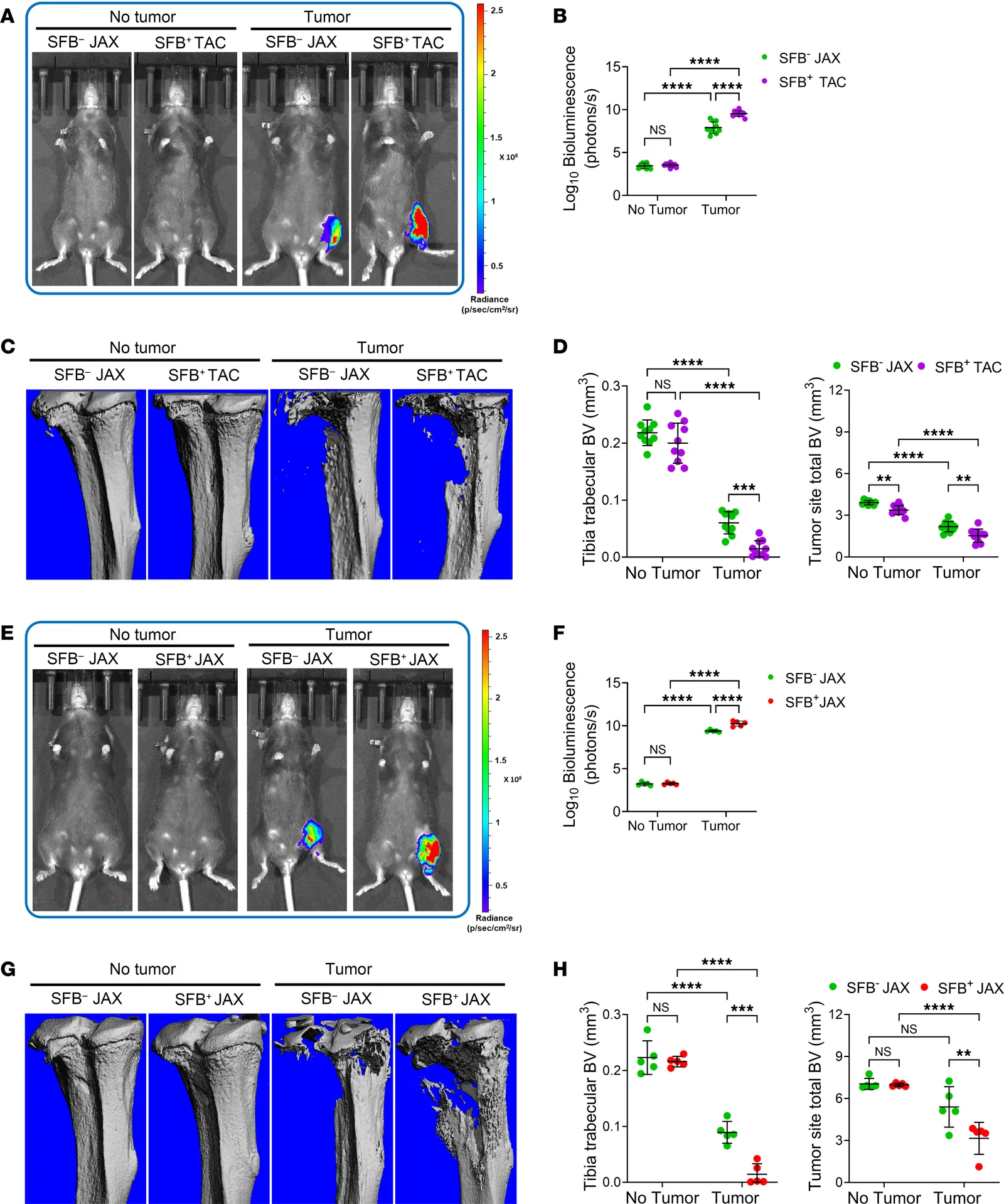
Effect of SFB on tumor bone growth. (**A** and **B**) Tumor bone growth in SFB^–^ JAX and SFB^+^ TAC mice assessed by luminescence. (**C**) Micro-CT scanning images of bone lesions in SFB^–^ JAX and SFB^+^ TAC mice. (**D**) Tibial trabecular BV and tumor site total BV in SFB^–^ JAX and SFB^+^ TAC mice. (**E** and **F**) Tumor bone growth in SFB^–^ JAX mice and SFB^+^ JAX mice assessed by luminescence. (**G**) Micro-CT scanning images of bone lesions in SFB^–^ JAX and SFB^+^ JAX mice. (**H**) Tibial trabecular BV and tumor site total BV in SFB^–^ JAX and SFB^+^ JAX mice. *n* = 5–10 mice per group. Data are expressed as mean ± SD. Luminescence tumor volume data were log_10_-transformed to ensure normality. All other data were normally distributed. All data were analyzed by 2-way ANOVA and post hoc tests applying Bonferroni’s correction for multiple comparisons. **P < 0.01, ***P < 0.001, ****P < 0.0001 compared with the indicated group.

**Figure 2:**
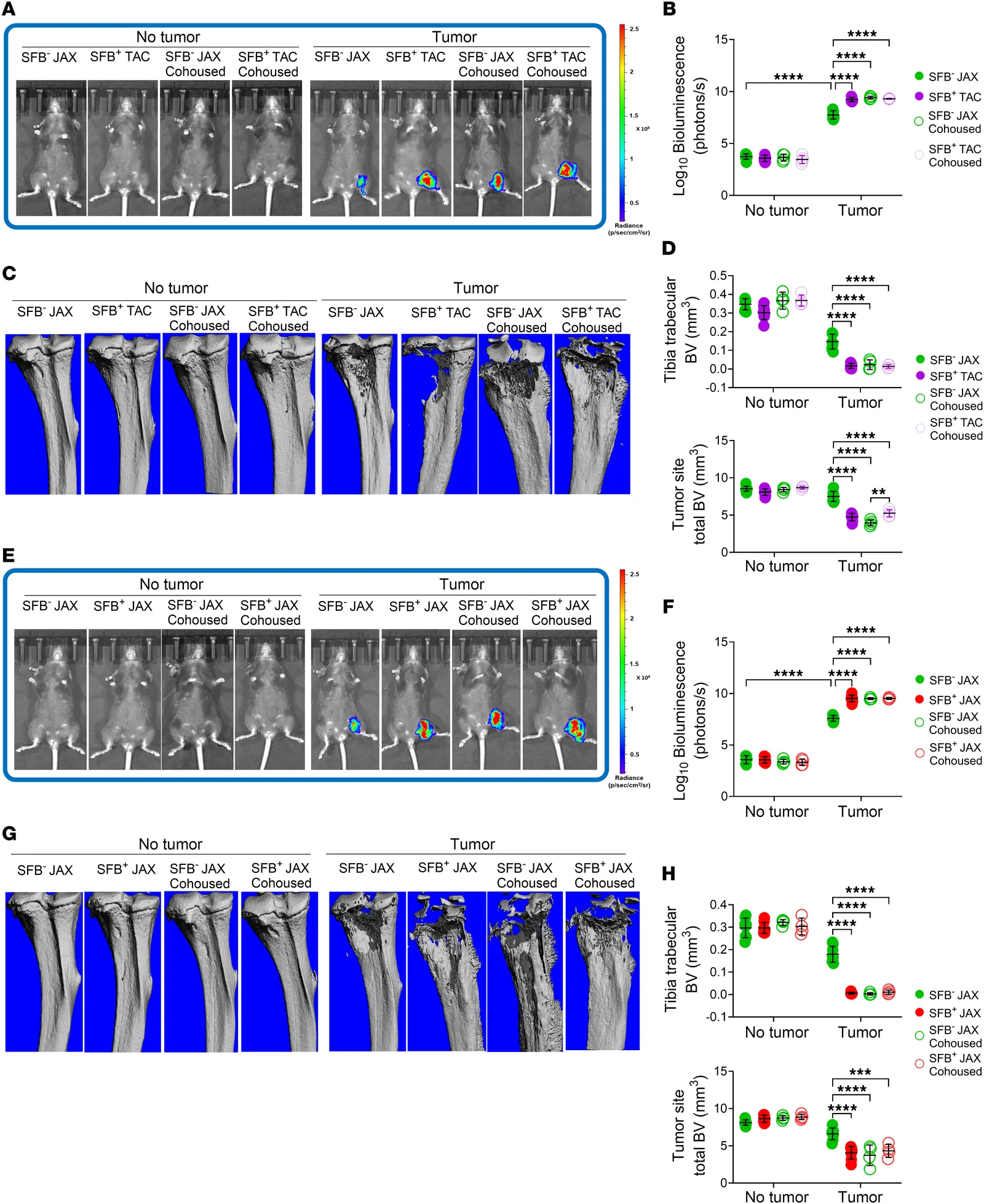
Effects of cohousing SFB^–^ and SFB^+^ mice on bone tumor growth. (**A** and **B**) Tumor bone growth assessment by luminescence in SFB^–^ JAX and SFB^+^ TAC mice housed separately or cohoused. (**C**) Micro-CT scanning images of bone lesions in SFB^–^ JAX and SFB^+^ TAC mice housed separately or cohoused. (**D**) Tibial trabecular BV and tumor site total BV in SFB^–^ JAX and SFB^+^ TAC mice housed separately or cohoused. (**E** and **F**) Tumor bone growth assessment by luminescence in SFB^–^ JAX mice and SFB^+^ JAX mice housed separately or cohoused. (**G**) Micro-CT scanning images of bone lesions in SFB^–^ JAX and SFB^+^ JAX mice housed separately or cohoused. (**H**) Tibial trabecular BV and tumor site total BV in SFB^–^ JAX and SFB^+^ JAX mice housed separately or cohoused. *n* = 8 mice per group. Data are expressed as mean ± SD. Luminescence tumor volume data were log_10_-transformed to ensure normality. All other data were normally distributed. All data were analyzed by 2-way ANOVA and post hoc tests applying Bonferroni’s correction for multiple comparisons. **P < 0.01, ***P < 0.001, ****P < 0.0001 compared with the indicated group.

**Figure 3:**
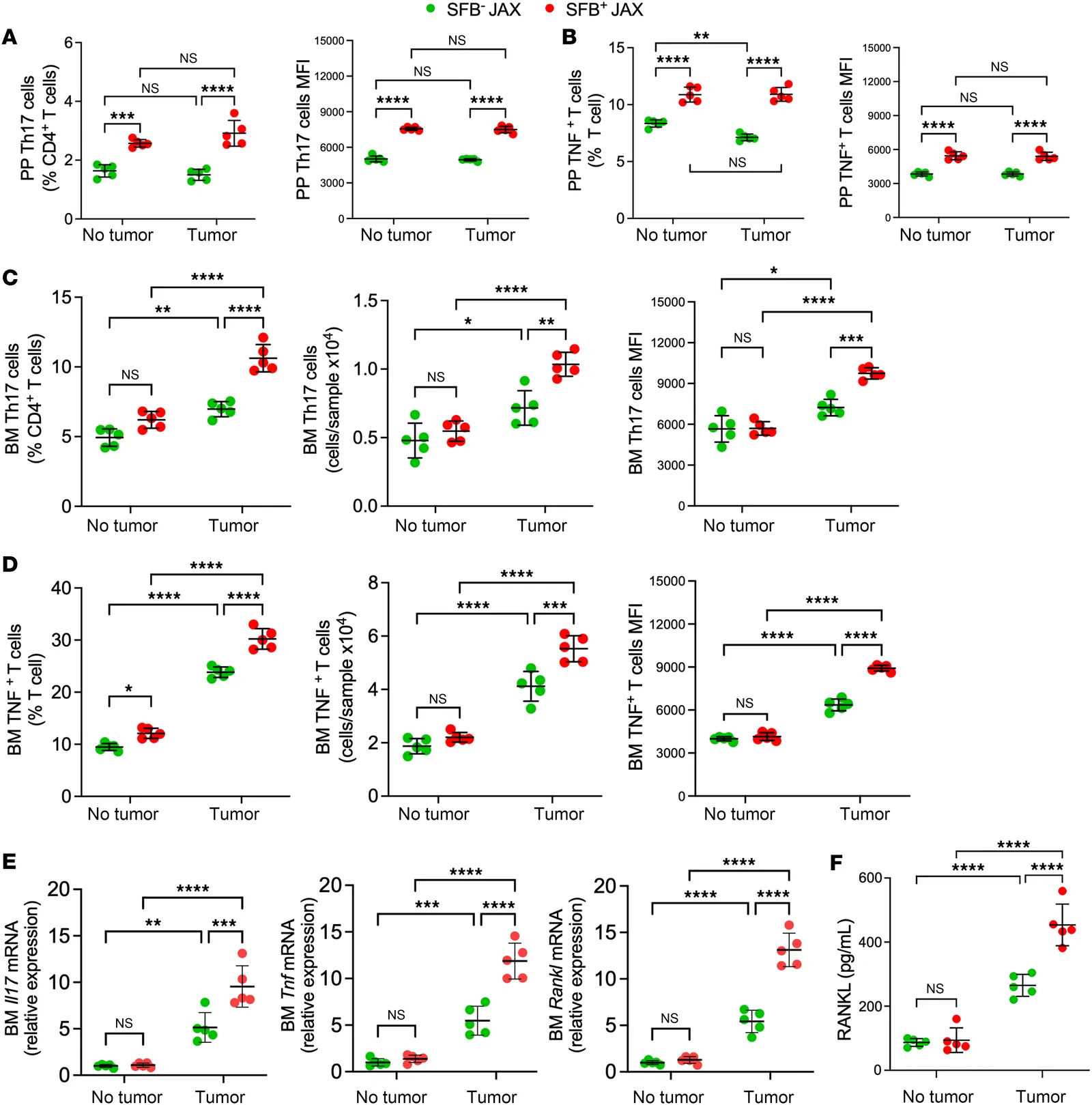
Effects of SFB on PP and BM Th17 cell and TNF^+^ T cell frequency and MFI and the levels of RANKL, IL-17, and TNF. (**A**) Relative frequency and MFI of PP Th17 cells. (**B**) Relative frequency and MFI of PP TNF^+^ T cells. (**C**) Relative frequency, absolute number, and MFI of BM Th17 cells. (**D**) Relative frequency, absolute number, and MFI of tumor site BM TNF^+^ T cells. (**E**) BM transcript levels of *Il17*, *Tnf*, and *Rankl*. (**F**) RANKL protein levels measured by ELISA. *n* = 5 mice per group. Data are expressed as mean ± SD. All data were normally distributed and were analyzed by 2-way ANOVA and post hoc tests applying Bonferroni’s correction for multiple comparisons. *P < 0.05, **P < 0.01, ***P < 0.001, ****P < 0.0001 compared with the indicated group.

**Figure 4:**
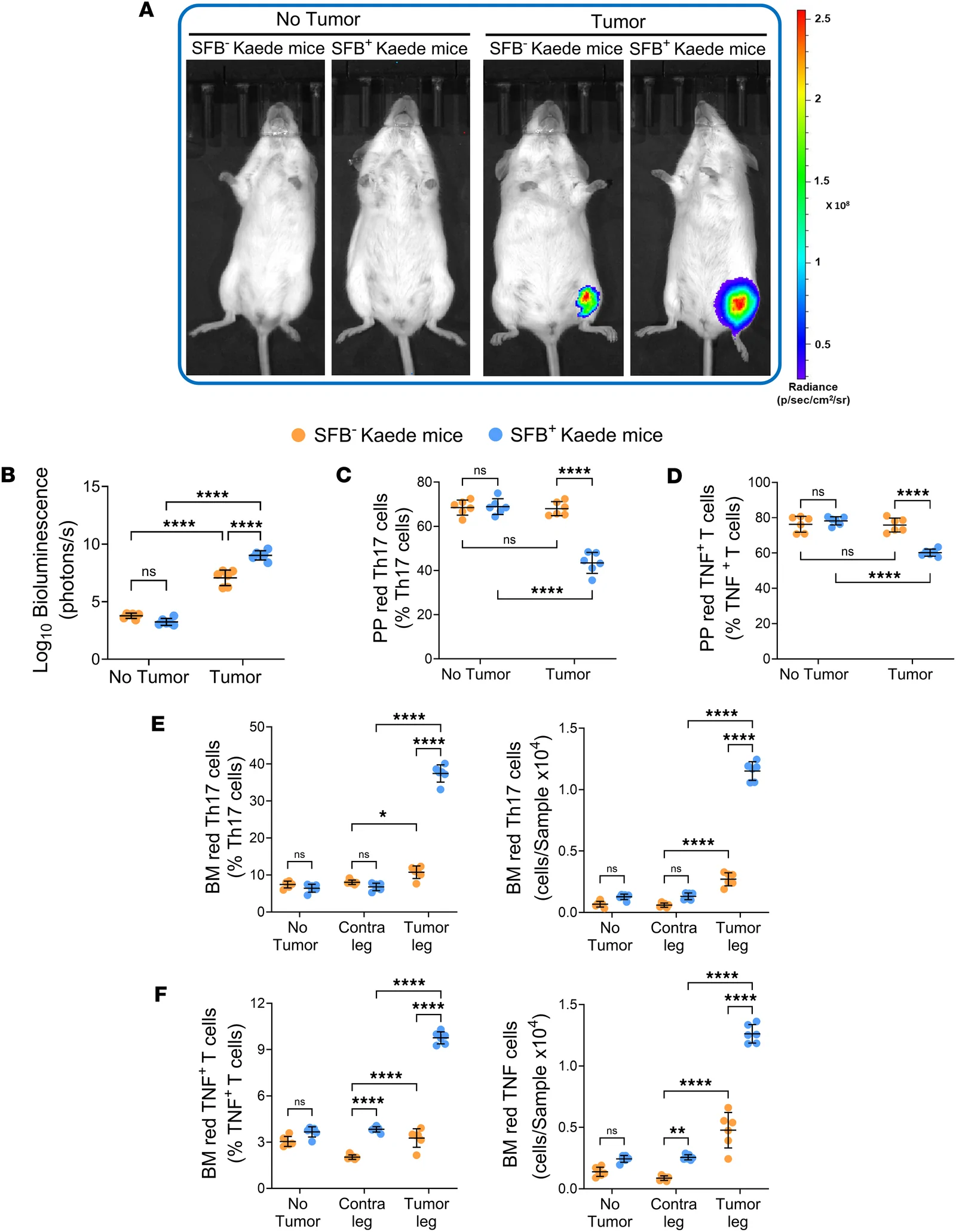
Effects of SFB on tumor growth and the migration of intestinal Th17 cells and TNF^+^ T cells to tumor site BM. Intratibial injections of PyMT cells were carried out in 10-week-old Kaede mice. The noninjected contralateral leg (Contra leg) was used as control. Nine days later, mice were subjected to surgical laparotomy to access the PPs in the distal small intestine. PP cells were photoconverted by exposure to a 390 nm light for 2 minutes. Mice were sacrificed 24 hours after photoconversion. (**A** and **B**) Tumor bone growth assessment by luminescence in SFB^–^ and SFB^+^ Kaede mice. (**C**) Relative frequency of PP red fluorescing Th17 cells. (**D**) Relative frequency of PP red fluorescing TNF^+^ T cells. (**E**) Relative frequency and absolute number of BM red fluorescing Th17 cells. (**F**) Relative frequency and absolute number of BM red fluorescing TNF^+^ T cells. *n* = 6 mice per group. Data are expressed as mean ± SD. Tumor volume, measured by bioluminescence, was log_10_-transformed to ensure normality. All other data were normally distributed. All data were analyzed by 2-way ANOVA and post hoc tests applying Bonferroni’s correction for multiple comparisons. *P < 0.05, **P < 0.01, ****P < 0.0001 compared with the indicated group.

**Figure 5:**
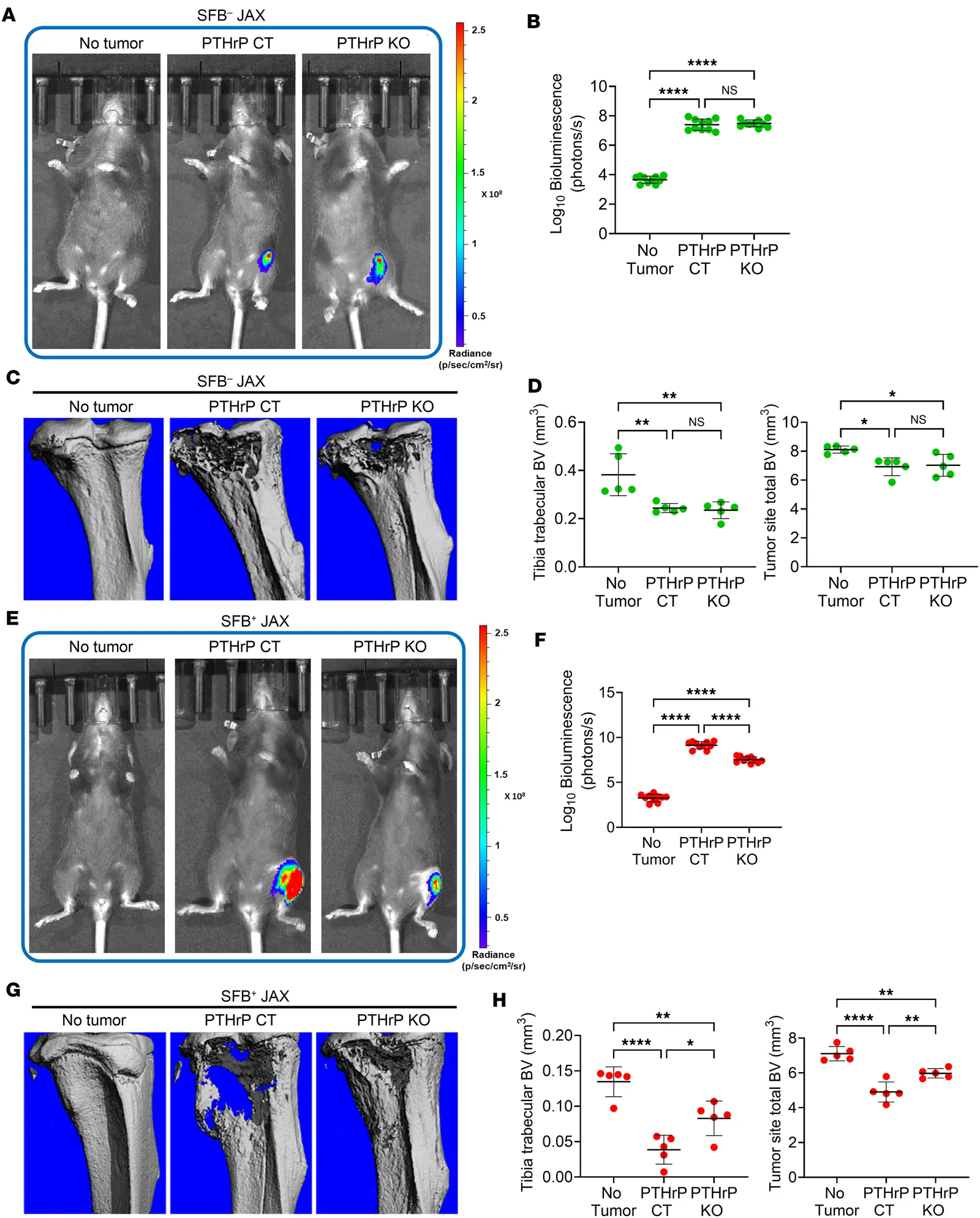
Bone tumor growth in SFB^–^ JAX mice and SFB^+^ JAX mice injected with PTHrP-KO PyMT cells. (**A** and **B**) Tumor bone growth in SFB^–^ JAX mice assessed by luminescence. (**C**) Micro-CT scanning images of bone lesions in SFB^–^ JAX mice. (**D**) Tibial trabecular BV and tumor site total BV in SFB^–^ JAX mice. (**E** and **F**) Tumor bone growth in SFB^+^ JAX mice assessed by luminescence. (**G**) Micro-CT scanning images of bone lesions in SFB^+^ JAX mice. (**H**) Tibial trabecular BV and tumor site total BV in SFB^+^ JAX mice. *n* = 5–10 mice per group. Data are expressed as mean ± SD. Luminescence data were log_10_-transformed to ensure normality. All other data were normally distributed. All data were analyzed by 1-way ANOVA and post hoc tests applying Bonferroni’s correction for multiple comparisons. *P < 0.05, **P < 0.01, ****P < 0.0001 compared with the indicated group.

**Figure 6:**
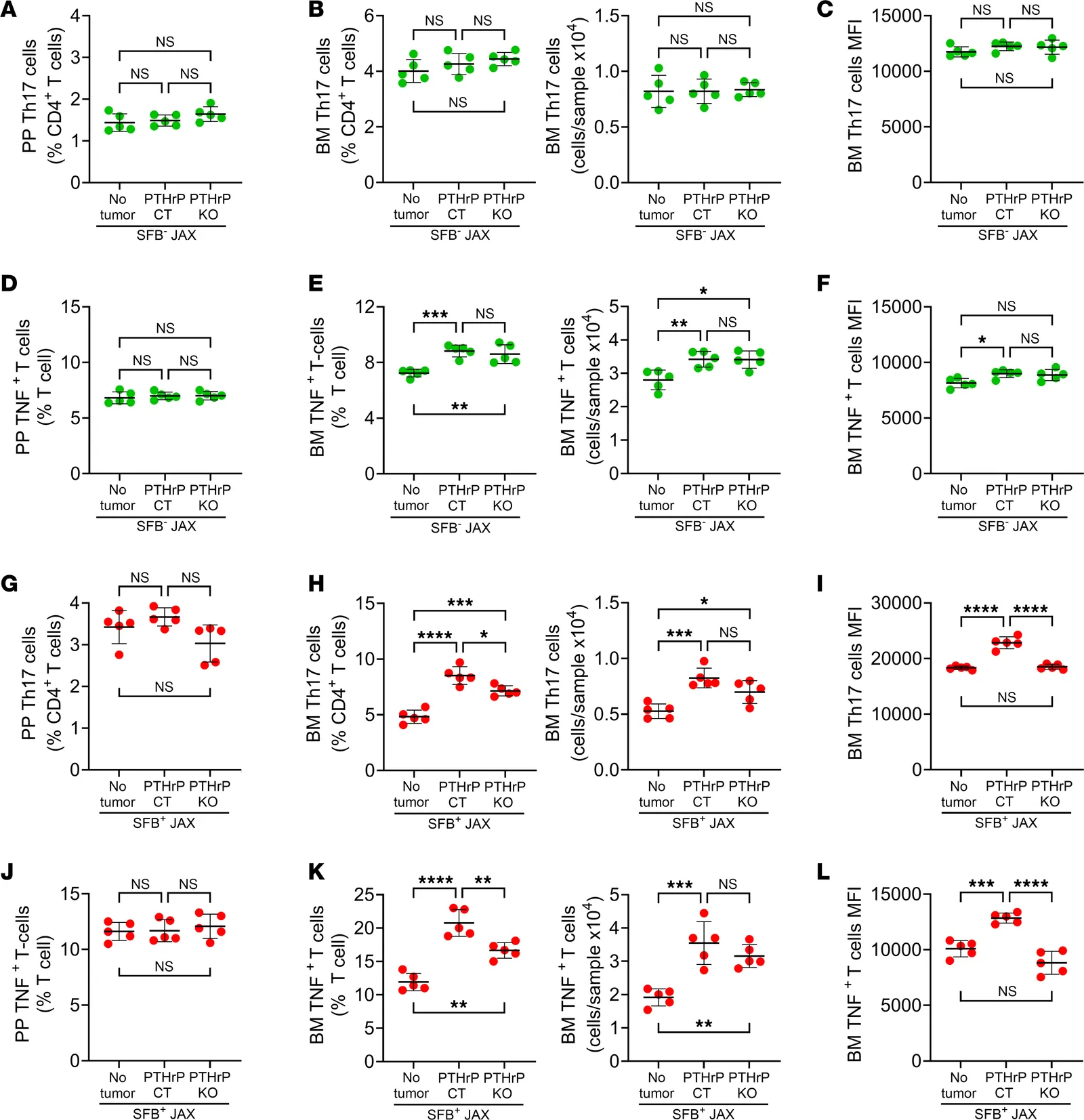
Effects of PTHrP deletion on Th17 cell and TNF^+^ T cell frequency and MFI in SFB^–^ JAX mice and SFB^+^ JAX mice. (**A**) Relative frequency of PP Th17 cells in JAX SFB^–^ mice. (**B**) Relative frequency and absolute number of tumor site BM Th17 cells in SFB^–^ JAX mice. (**C**) BM Th17 cell MFI in SFB^–^ JAX mice. (**D**) Relative frequency of PP TNF^+^ T cells in SFB^–^ JAX mice. (**E**) Relative frequency and absolute number of tumor site BM TNF^+^ T cells in SFB^–^ JAX mice. (**F**) Tumor site BM TNF^+^ T cell MFI in SFB^–^ JAX mice. (**G**) Relative frequency of PP Th17 cells in SFB^+^ JAX mice. (**H**) Relative frequency and absolute number of tumor site BM Th17 cells in SFB^+^ JAX mice. (**I**) Tumor site BM Th17 cell MFI in SFB^+^ JAX mice. (**J**) Relative frequency of PP TNF^+^ T cells in SFB^+^ JAX mice. (**K**) Relative frequency and absolute number of tumor site BM TNF^+^ T cells in SFB^+^ JAX mice. (**L**) Tumor site BM TNF^+^ T cell MFI in SFB^+^ JAX mice. *n* = 5 mice per group. Data are expressed as mean ± SD. All data were normally distributed and were analyzed by 1-way ANOVA and post hoc tests applying Bonferroni’s correction for multiple comparisons. *P < 0.05, **P < 0.01, ***P < 0.001, ****P < 0.0001 compared with the indicated group.

**Figure 7:**
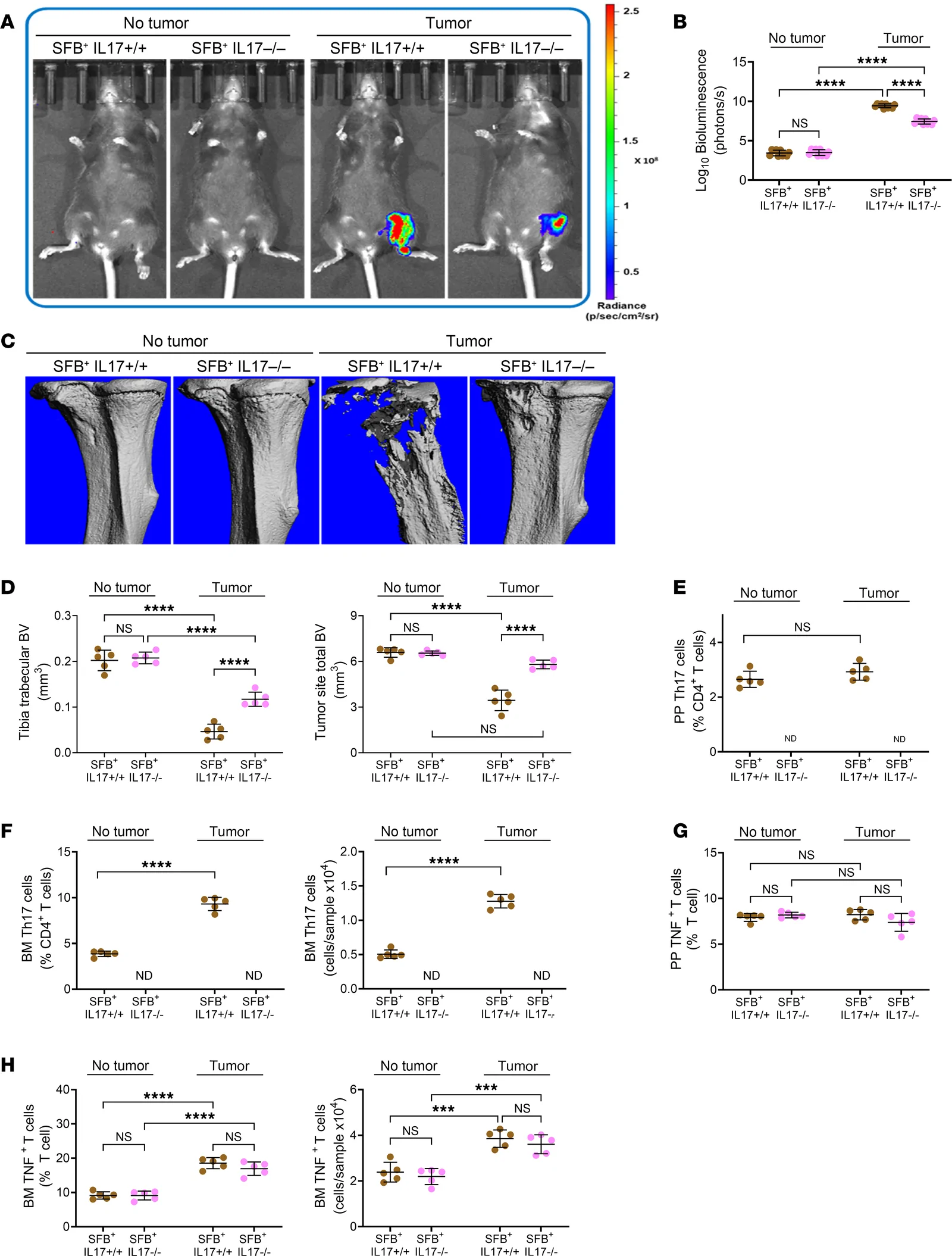
Global deletion of IL-17 prevents the expansion of Th17 cells at the tumor site induced by intratibial injection of PyMT breast cancer cells, leading to reduced tumor growth. (**A** and **B**) Tumor bone growth in SFB^+^ IL-17^–/–^ mice and control littermates assessed by luminescence. (**C**) Micro-CT scanning images of bone lesions in SFB^+^ IL-17^–/–^ mice and control littermates. (**D**) Tibial trabecular BV and tumor site total BV in SFB^+^ IL-17^–/–^ mice and control littermates. (**E**) Relative frequency of PP Th17 cells. (**F**) Relative frequency and absolute number of tumor site BM Th17 cells. (**G**) Relative frequency of PP TNF^+^ T cells. (**H**) Relative frequency and absolute number of tumor site BM TNF^+^ T cells. *n* = 5–10 mice per group. Data are expressed as mean ± SD. Luminescence data were log_10_-transformed to ensure normality. All other data were normally distributed. All data were analyzed by either 2-tailed *t* test or 2-way ANOVA and post hoc tests applying Bonferroni’s correction for multiple comparisons. ***P < 0.001, ****P < 0.0001 compared with the indicated group.

**Figure 8:**
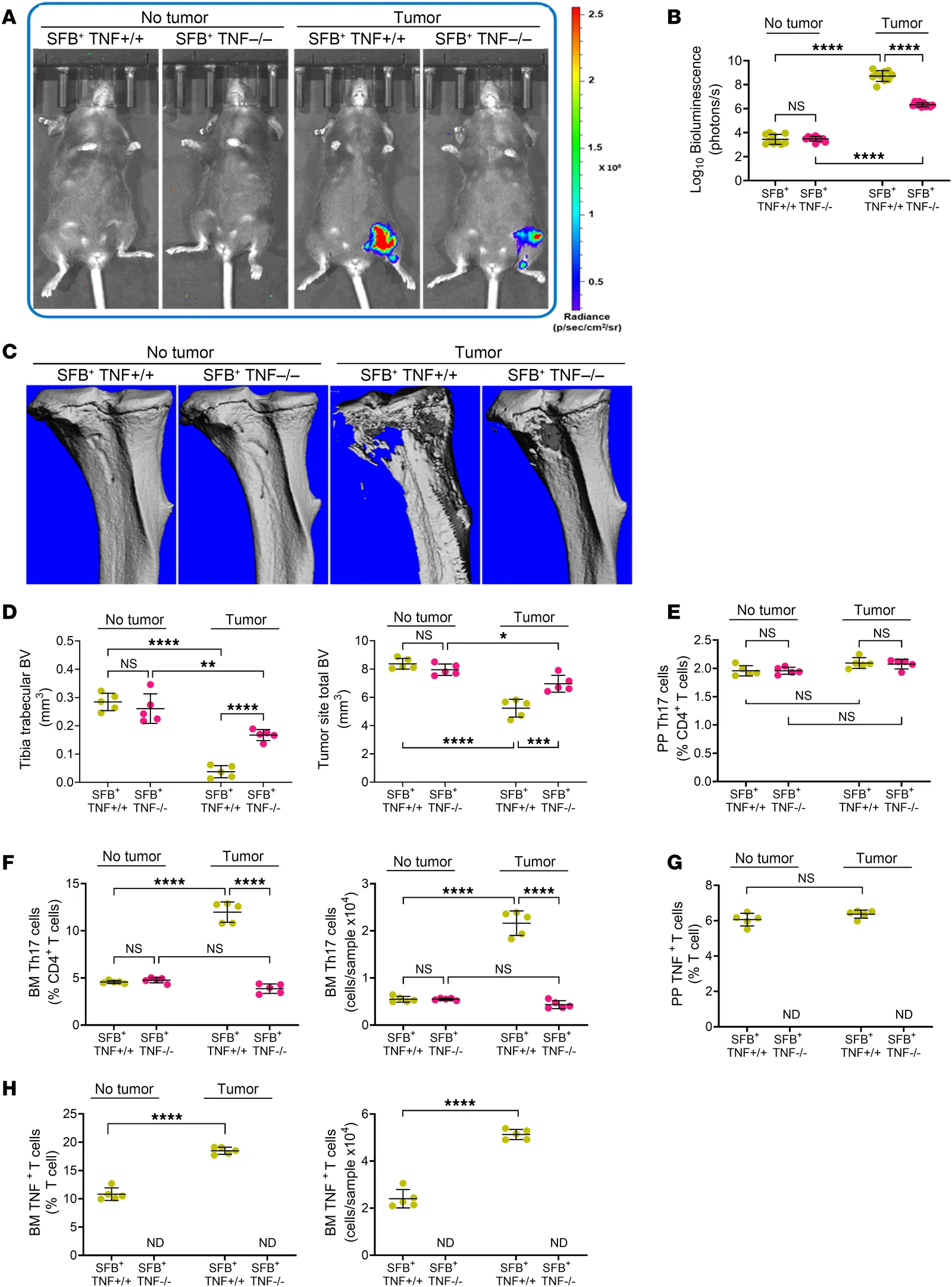
Global deletion of TNF prevents the expansion of Th17 cells and TNF^+^ T cells at the tumor site induced by intratibial injection of PyMT breast cancer cells, leading to reduced tumor growth. (**A** and **B**) Tumor bone growth in SFB^+^ TNF^–/–^ mice and control littermates assessed by luminescence. (**C**) Micro-CT scanning images of bone lesions in SFB^+^ TNF^–/–^ mice and control littermates. (**D**) Tibial trabecular BV and tumor site total BV in SFB^+^ TNF^–/–^ mice and control littermates. (**E**) Relative frequency of PP Th17 cells. (**F**) Relative frequency and absolute number of tumor site BM Th17 cells. (**G**) Relative frequency of PP TNF^+^ T cells. (**H**) Relative frequency and absolute number of tumor site BM TNF^+^ T cells. *n* = 5–10 mice per group. Data are expressed as mean ± SD. Luminescence data were log_10_-transformed to ensure normality. All other data were normally distributed. All data were analyzed by either 2-tailed *t* test or 2-way ANOVA and post hoc tests applying Bonferroni’s correction for multiple comparisons. *P < 0.05, **P < 0.01, ***P < 0.001, ****P < 0.0001 compared with the indicated group.

## Version 1 10/22/2026

Electronic publication
