# Supplementary material for: PTHrP induces breast cancer bone metastases via microbial-dependent expansion of TNF/IL-17–producing T cells
Source: JCI Insight. 2026 Oct 22;11(20):e207567. doi: 10.1172/jci.insight.207567 (PMC13726555; doi:10.1172/jci.insight.207567)
Supplement: Supplementary file 1 [file jciinsight-11-207567-s212.pdf]

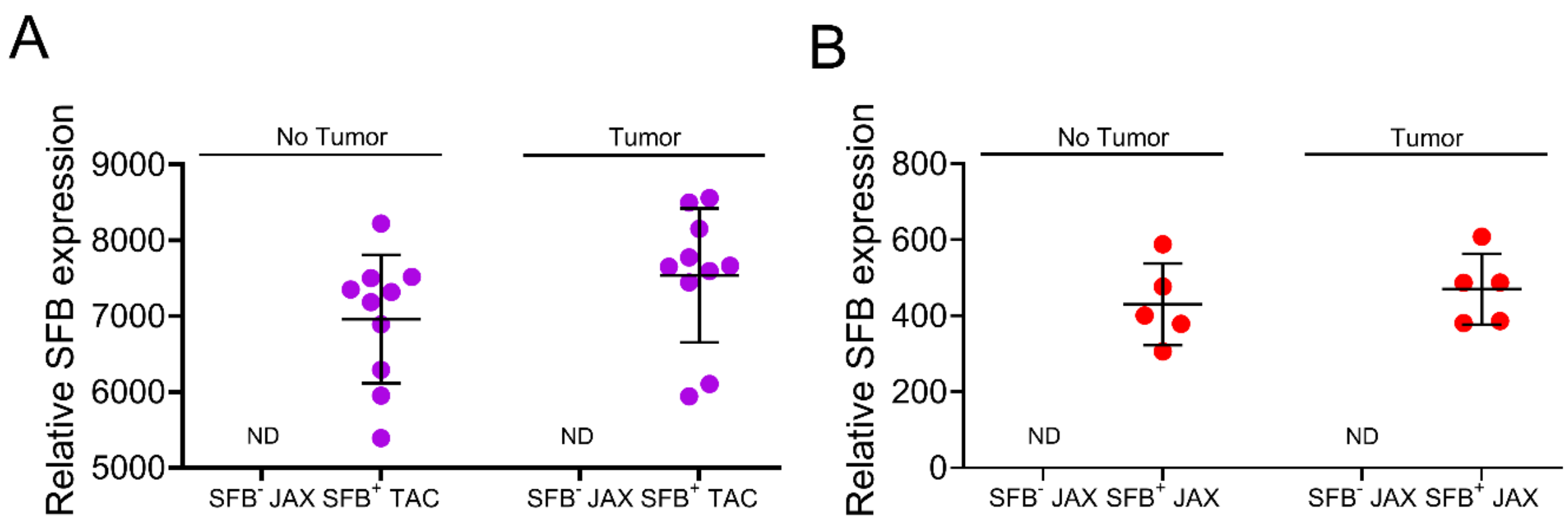

Supplemental Figure 1. PCR analysis of total bacterial DNA targeting SFB-specific 16S rRNA genes in stool samples from JAX and TAC mice. (A) Relative SFB expression in SFB^- JAX and SFB^+ TAC mice. **(B)** Relative SFB expression in SFB^- JAX and SFB^+ JAX mice group. Data are expressed as Mean ± SD. n = 5-10 mice per group. ND = non detectable.

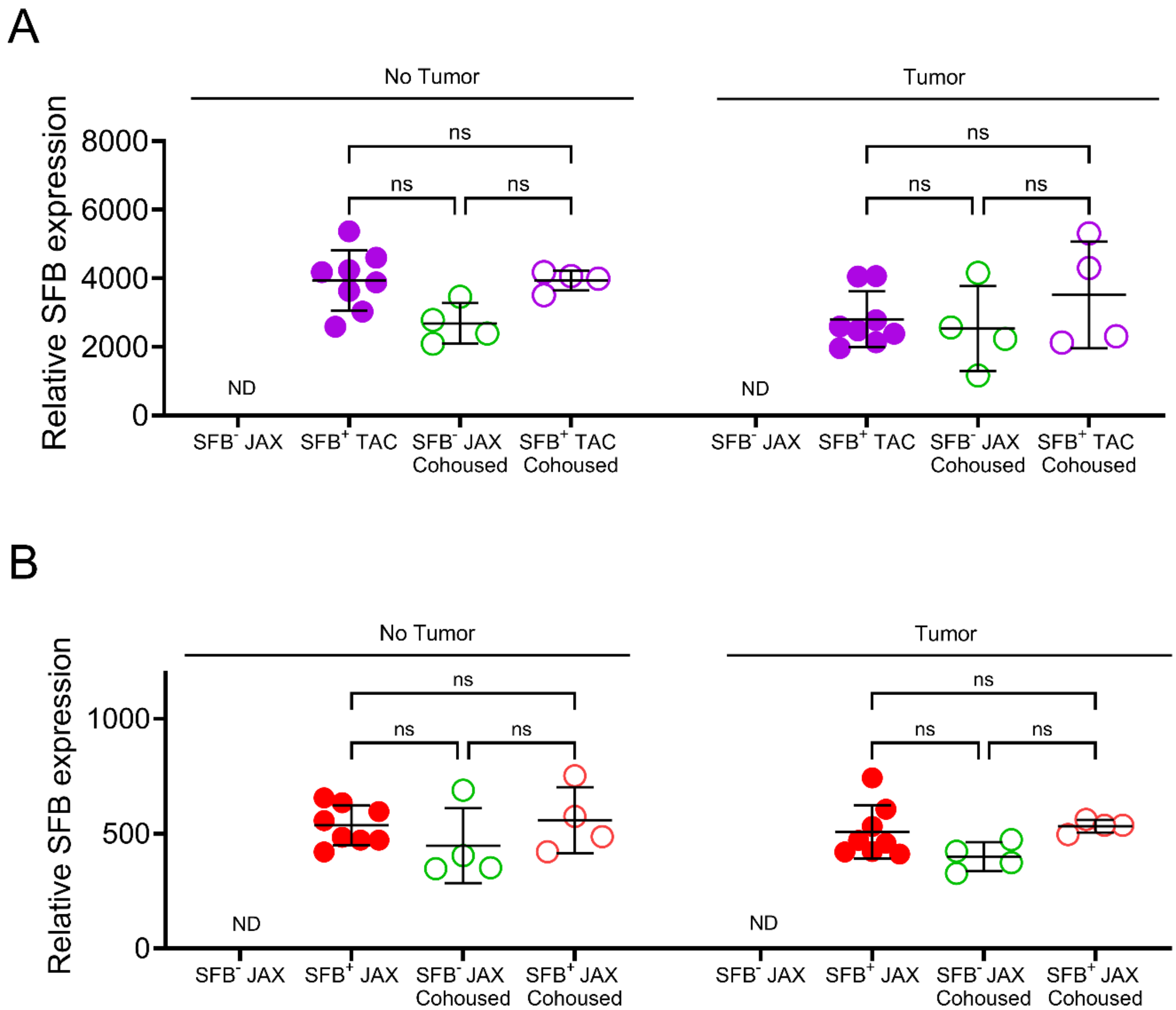

Supplemental Figure 2. PCR analysis of total bacterial DNA targeting SFB-specific 16S rRNA genes in stool samples from single housed and cohoused mice. (A) Single housed and cohoused SFB$^-$ JAX and SFB$^+$ TAC mice. **(B)** Single housed and cohoused SFB$^-$ JAX and SFB$^+$ JAX mice. n = 4-8 mice per group. Data are expressed as mean ± SD. The normally distributed data were analyzed by two-way ANOVA and post hoc tests applying Bonferroni's correction for multiple comparisons. ND = non detectable. ns = not significant.

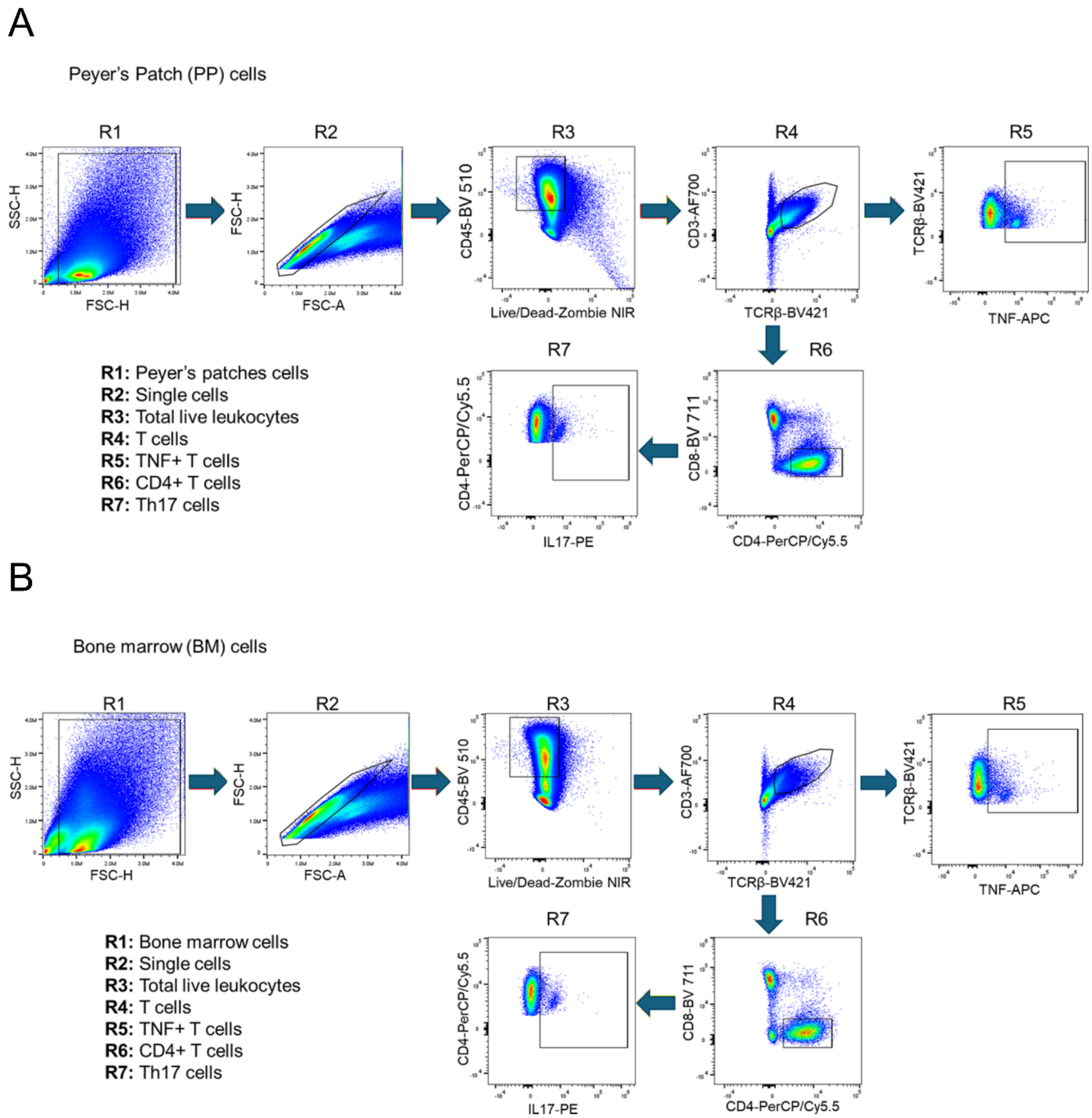

Supplemental Figure 3. Gating strategy used to identify PP (A) and BM (B) TNF^{+} T cells and Th17 cells. Single-cell suspensions from PP and BM were obtained after red blood cell lysis. These were subsequently stained with antibodies targeting the specified antigens, along with a live/dead cell dye. A representative flow cytometry plot is shown, with the sequential gating regions labeled R1 to R7.

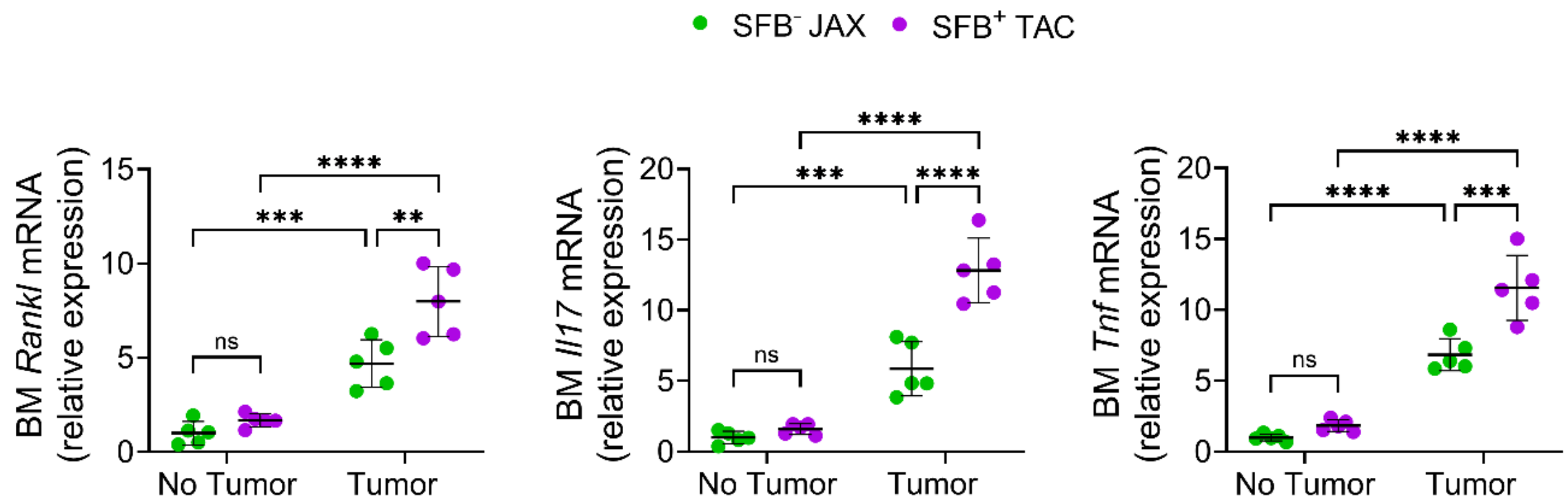

Supplemental Figure 4. Tumor site BM *Rankl*, *Il17*, and *Tnf* mRNA transcript levels in SFB$^-$ JAX and SFB$^+$ TAC mice. Data are expressed as mean ± SD. n = 5 mice per group. All data were normally distributed and were analyzed by two-way ANOVA and post hoc tests applying Bonferroni's correction for multiple comparisons. ** = P < 0.01, *** = P < 0.001, **** = P < 0.0001, ns = not significant compared with the indicated group.

Pro-tumorigenic Th17 cells

Bone marrow cells

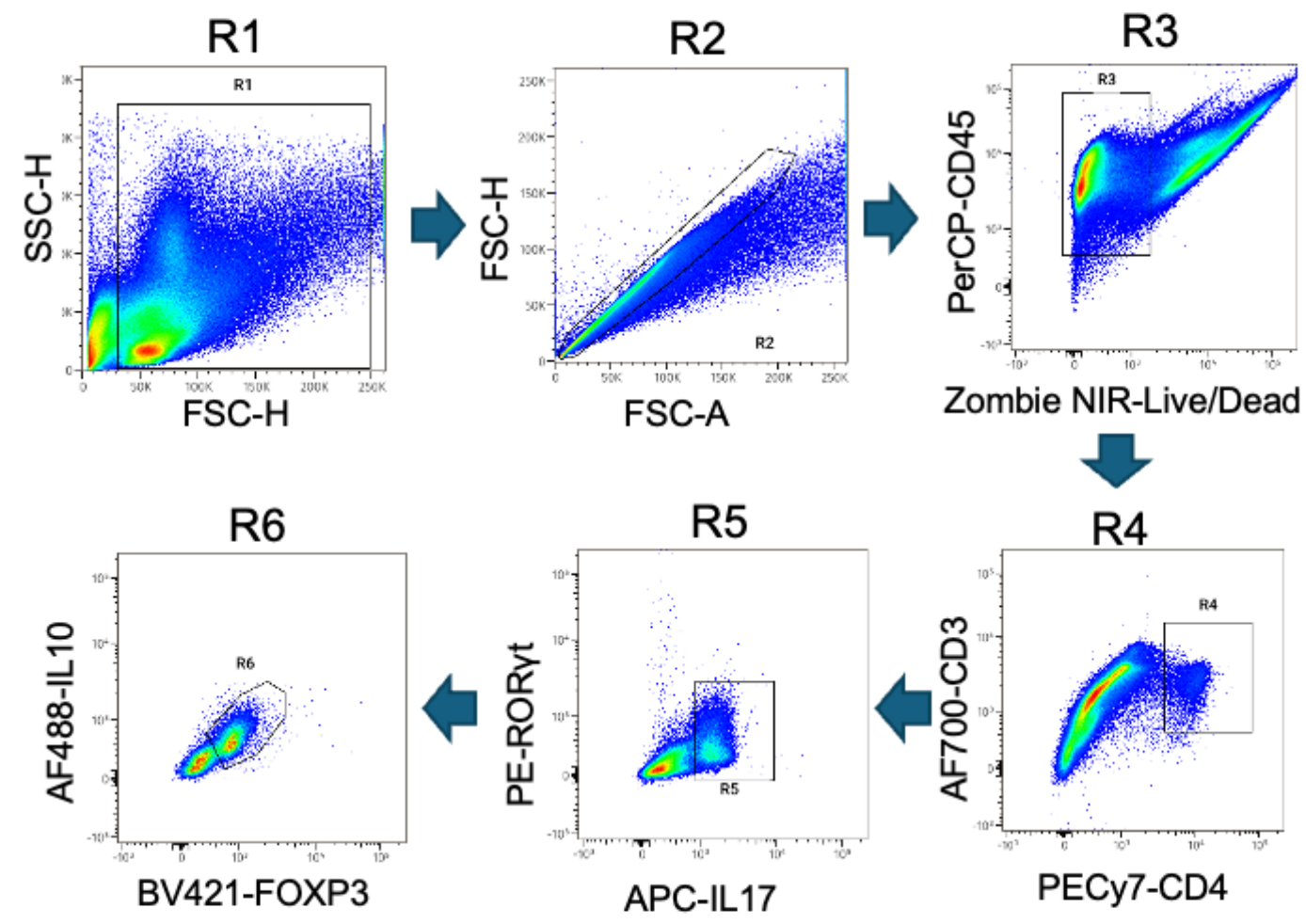

R1: Bone marrow cells
R2: Single cells
R3: Total live leukocytes
R4: CD4+ T cells
R5: Th17 cells
R6: FOXP3+ IL-10+ Th17 cells

Anti-tumorigenic Th17 cells

Bone marrow cells

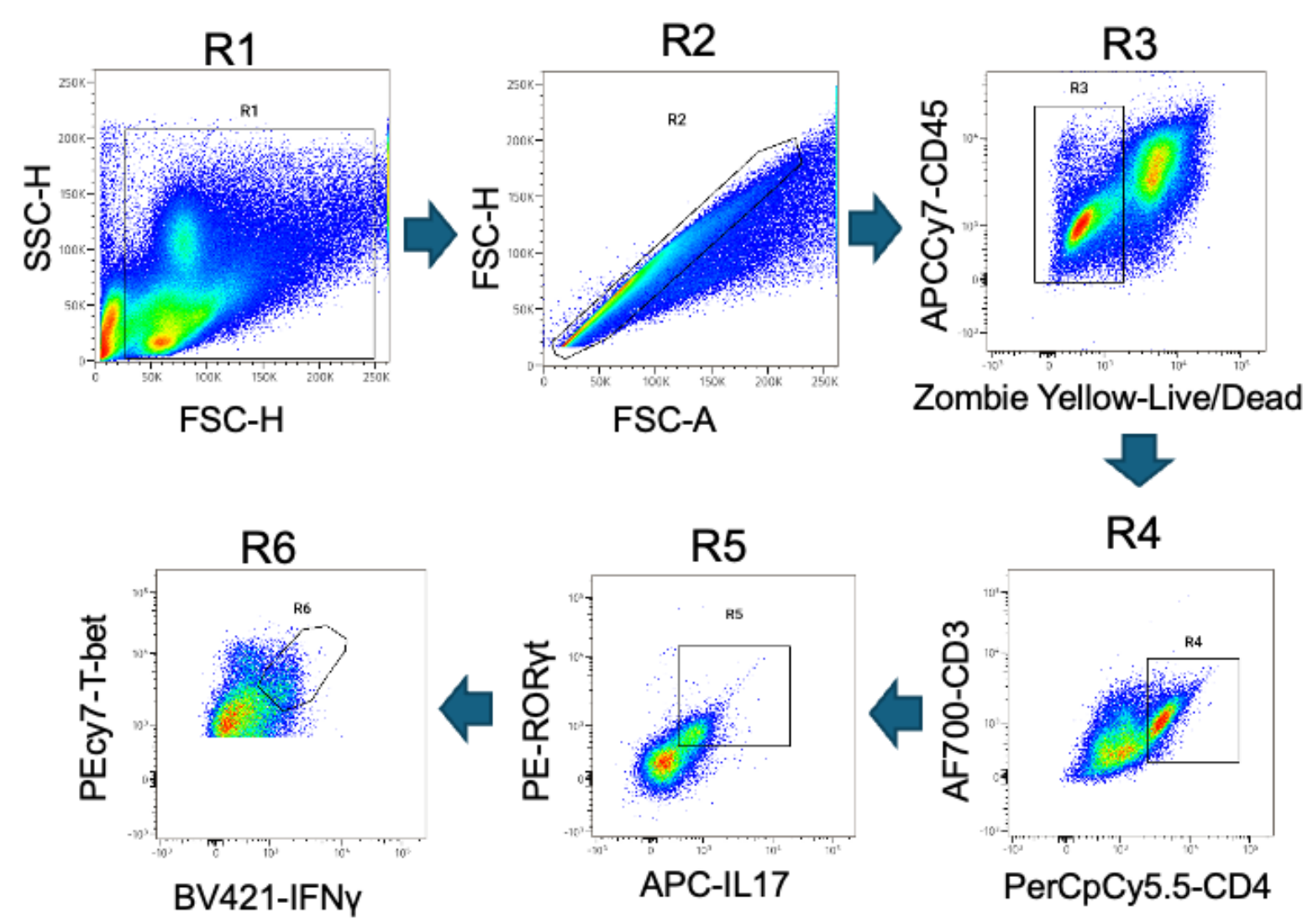

R1: Bone marrow cells
R2: Single cells
R3: Total live leukocytes
R4: CD4+ T cells
R5: Th17 cells
R6: T-bet+ IFNγ+ Th17 cells

Supplemental Figure 5. Gating strategy used to identify pro- and anti- tumorigenic Th17 cells. Single-cell suspensions from PP and BM were obtained after red blood cell lysis. These were subsequently stained with antibodies targeting the specified antigens, along with a live/dead cell dye. Representative flow cytometry plot shown, with the sequential gating regions labeled R1 to R6.

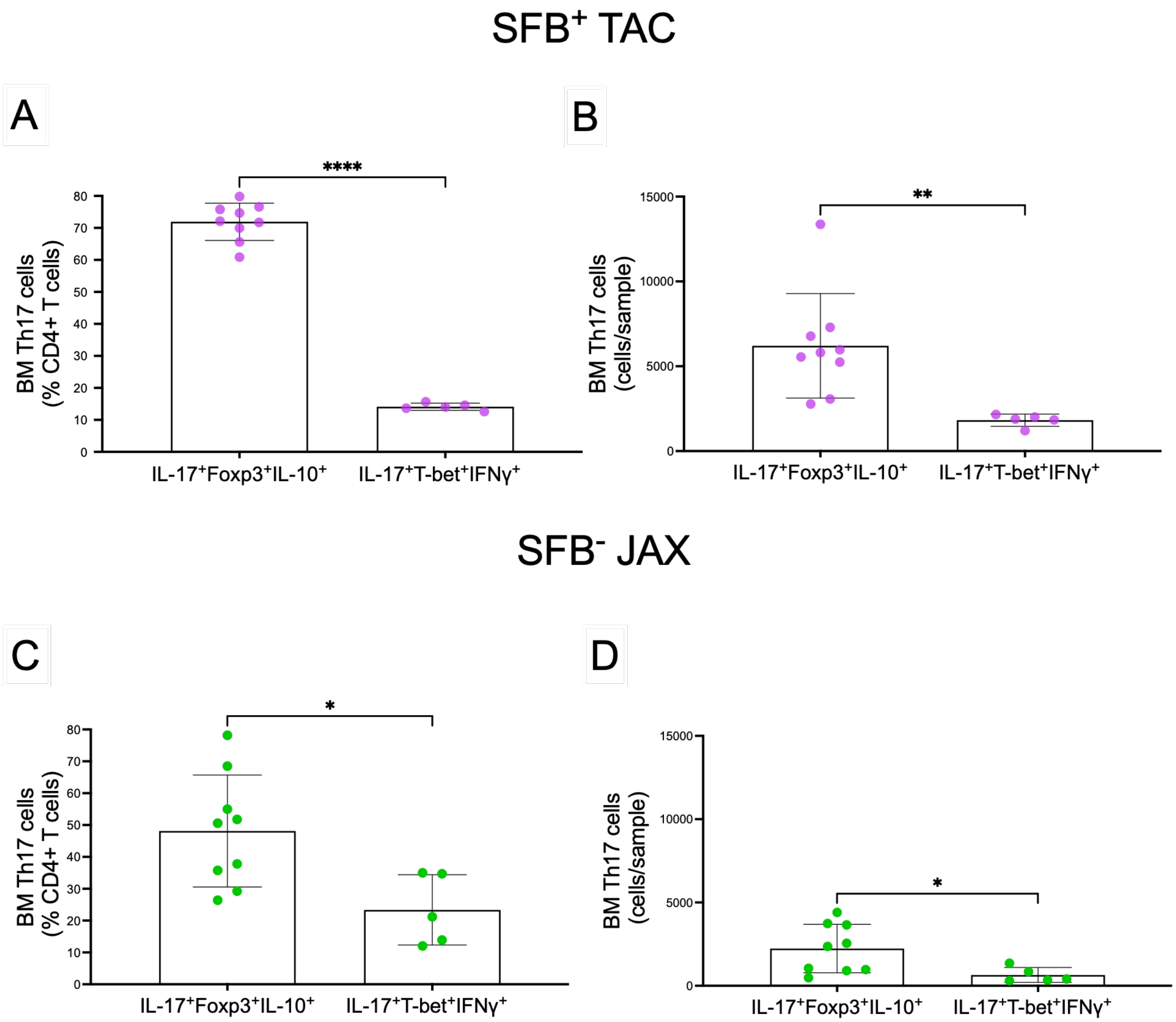

Supplemental Figure 6. Relative and absolute frequency of pro- and anti-tumorigenic Th17 cells in tumor site BM. (A) Relative frequency of pro-tumorigenic regulatory Th17 cells and anti-tumorigenic Th1-like Th17 cells in SFB$^+$ TAC mice. **(B)** Absolute number of pro-tumorigenic regulatory Th17 cells and anti-tumorigenic Th1-like Th17 cells in SFB$^+$ TAC mice. **(C)** Relative frequency of pro-tumorigenic regulatory Th17 cells and anti-tumorigenic Th1-like Th17 cells in SFB$^-$ JAX mice. **(D)** Absolute number of pro-tumorigenic regulatory Th17 cells and anti-tumorigenic Th1-like Th17 cells in SFB$^-$ JAX mice. n = 5-9 mice per group. Data are expressed as mean ± SD. Data were compared by unpaired 2-tailed t tests. * = $P < 0.05$, ** = $P < 0.01$, and **** = $P < 0.0001$ compared with the indicated group.

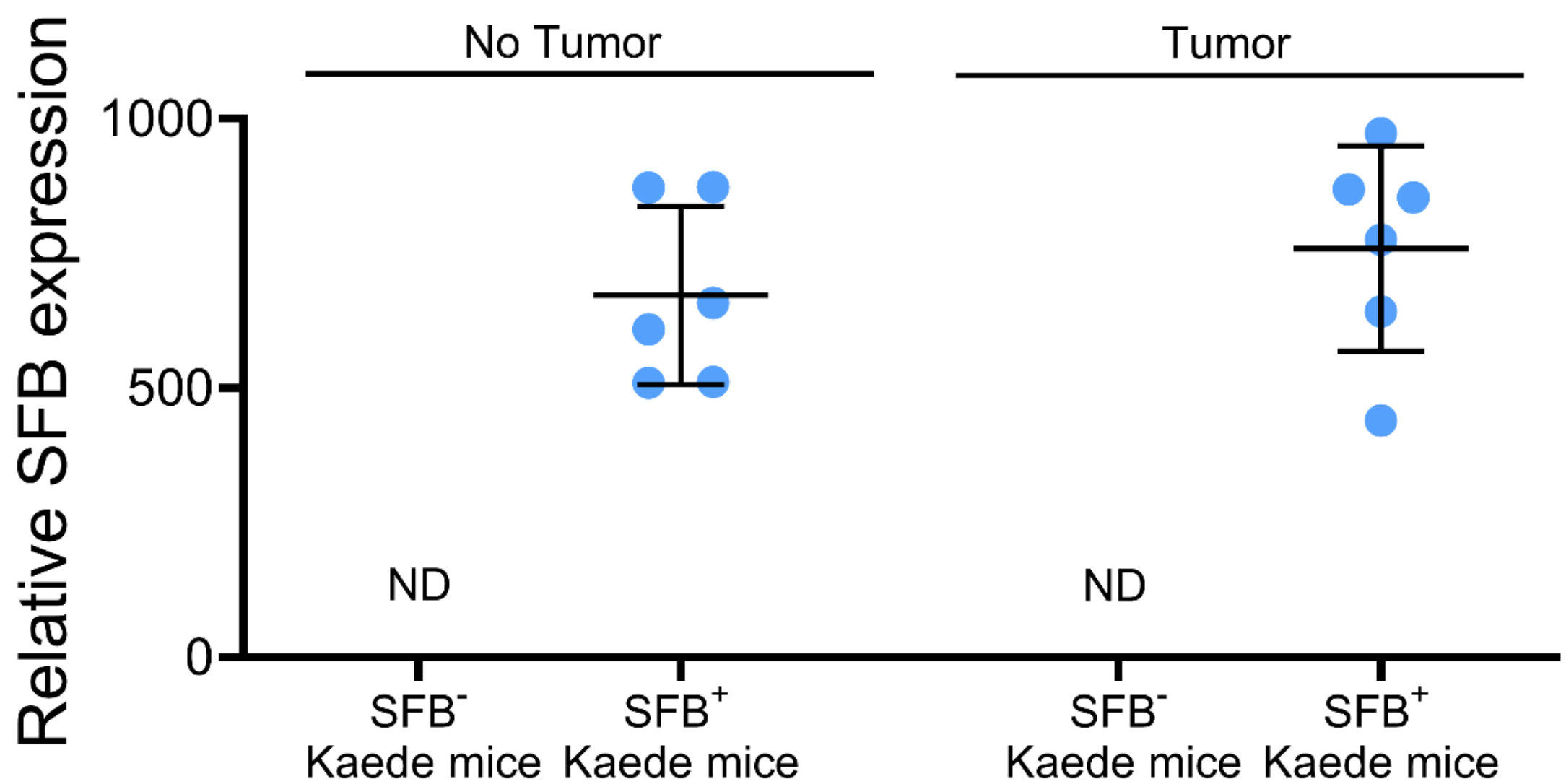

Supplemental Figure 7. PCR analysis of total bacterial DNA targeting SFB-specific 16S rRNA genes in stool samples from Kaede mice. n = 6 mice per group. Data are expressed as mean ± SD. ND = non detectable.

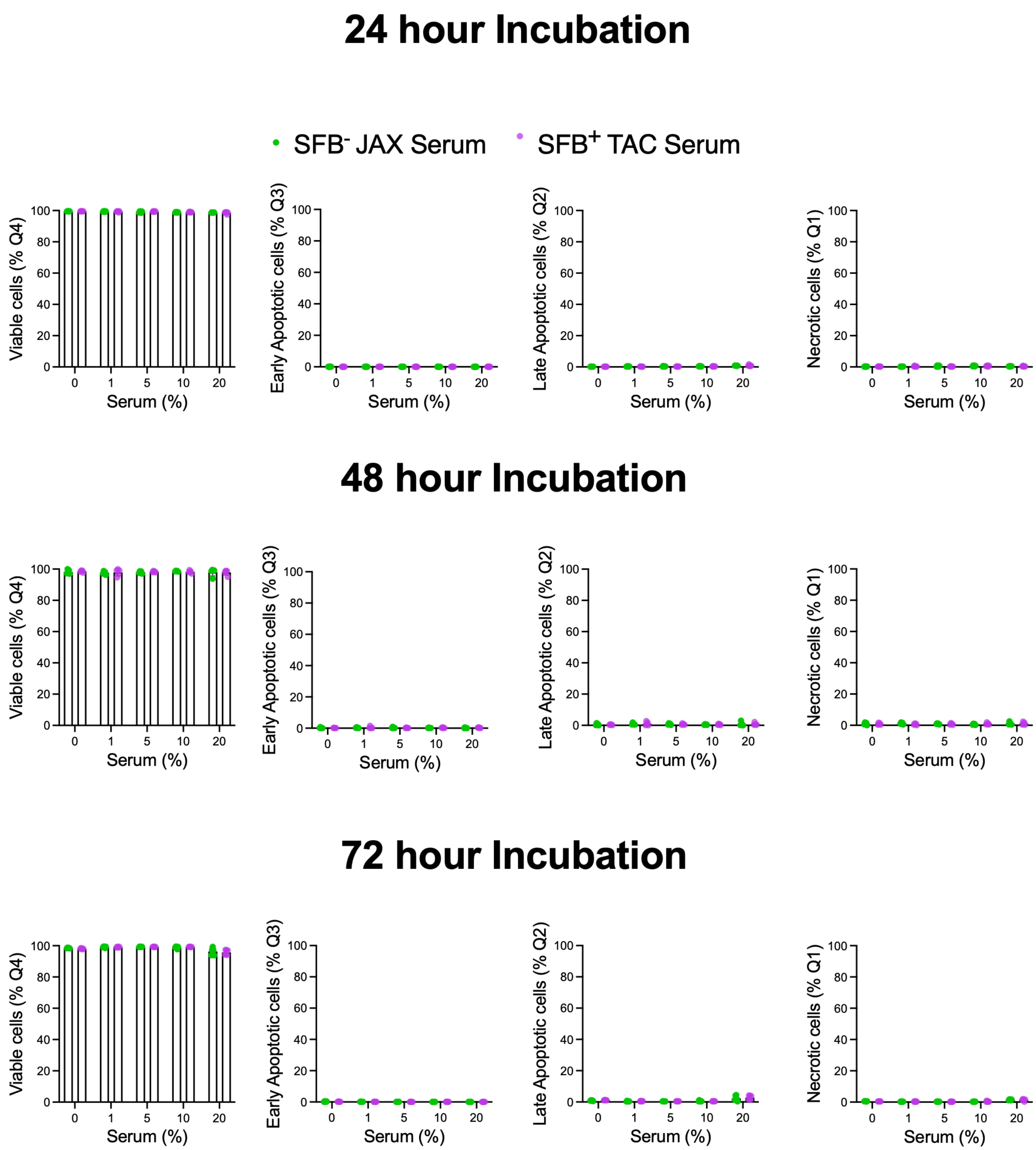

Supplemental Figure 8. Effects of serum from SFB$^+$ or SFB$^-$ mice on PyMT cell proliferation and survival. PyMT cells were treated with different concentrations of Serum (0, 0.1, 5, 10, or 20 %) and analyzed using flow cytometry after staining with Annexin V-FITC and PI at 24 h, 48 h, and 72 h incubation. Four populations were indicated as necrotic dead cells (Q1), late apoptotic cells (Q2), early apoptotic cells (Q3), and viable cells (Q4). n = 5-6 samples per group. Data are expressed as mean ± SD. Data were compared by two-way ANOVA and post hoc tests applying Bonferroni's correction for multiple comparisons. Non-significant comparisons not shown.

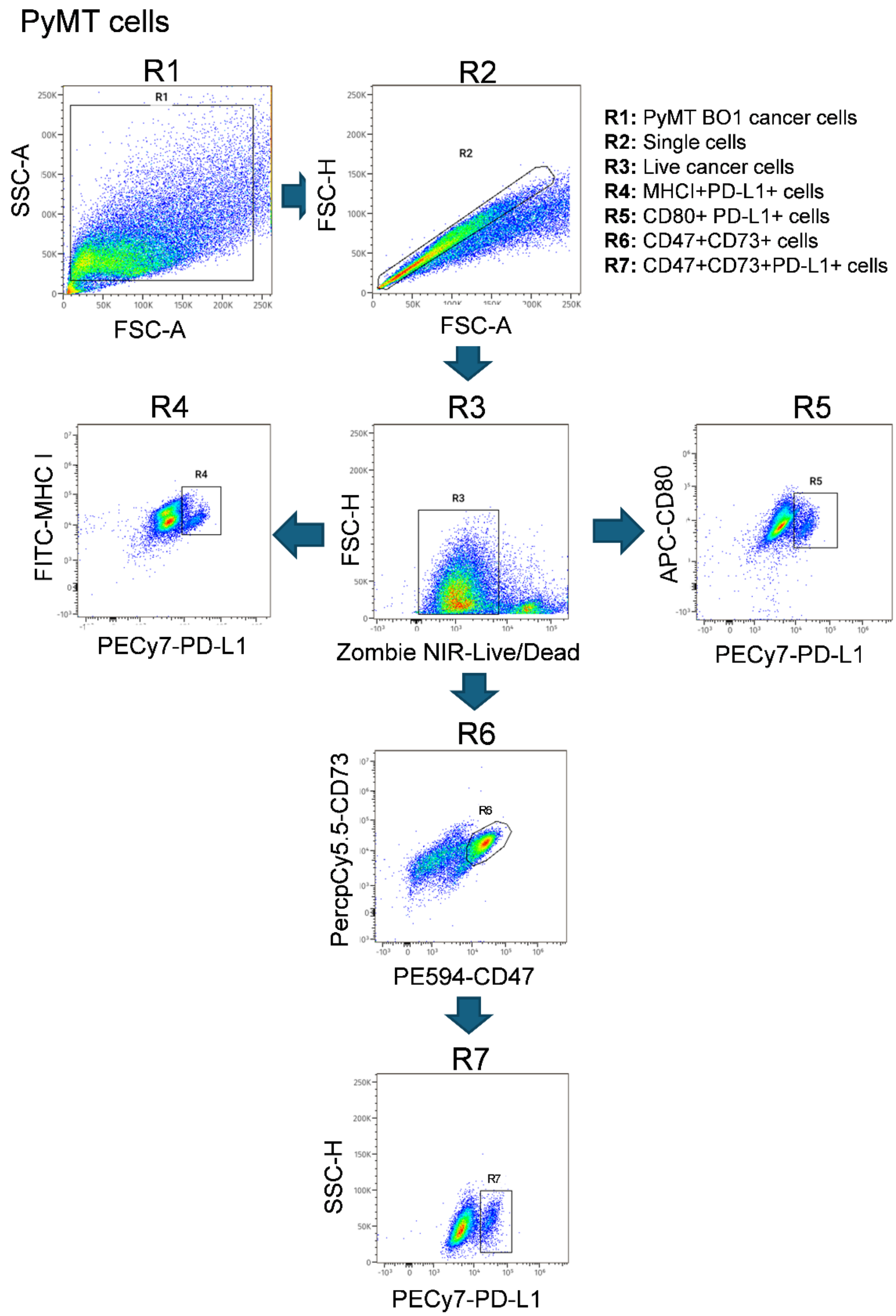

Supplemental Figure 9. Gating strategy used to identify immune evasive phenotypes of PyMT cells. Single-cell suspensions of PyMT cells were prepared. These were subsequently stained with antibodies targeting the specified antigens, along with a live/dead cell dye. Representative flow cytometry plot shown, with the sequential gating regions labeled R1 to R7.

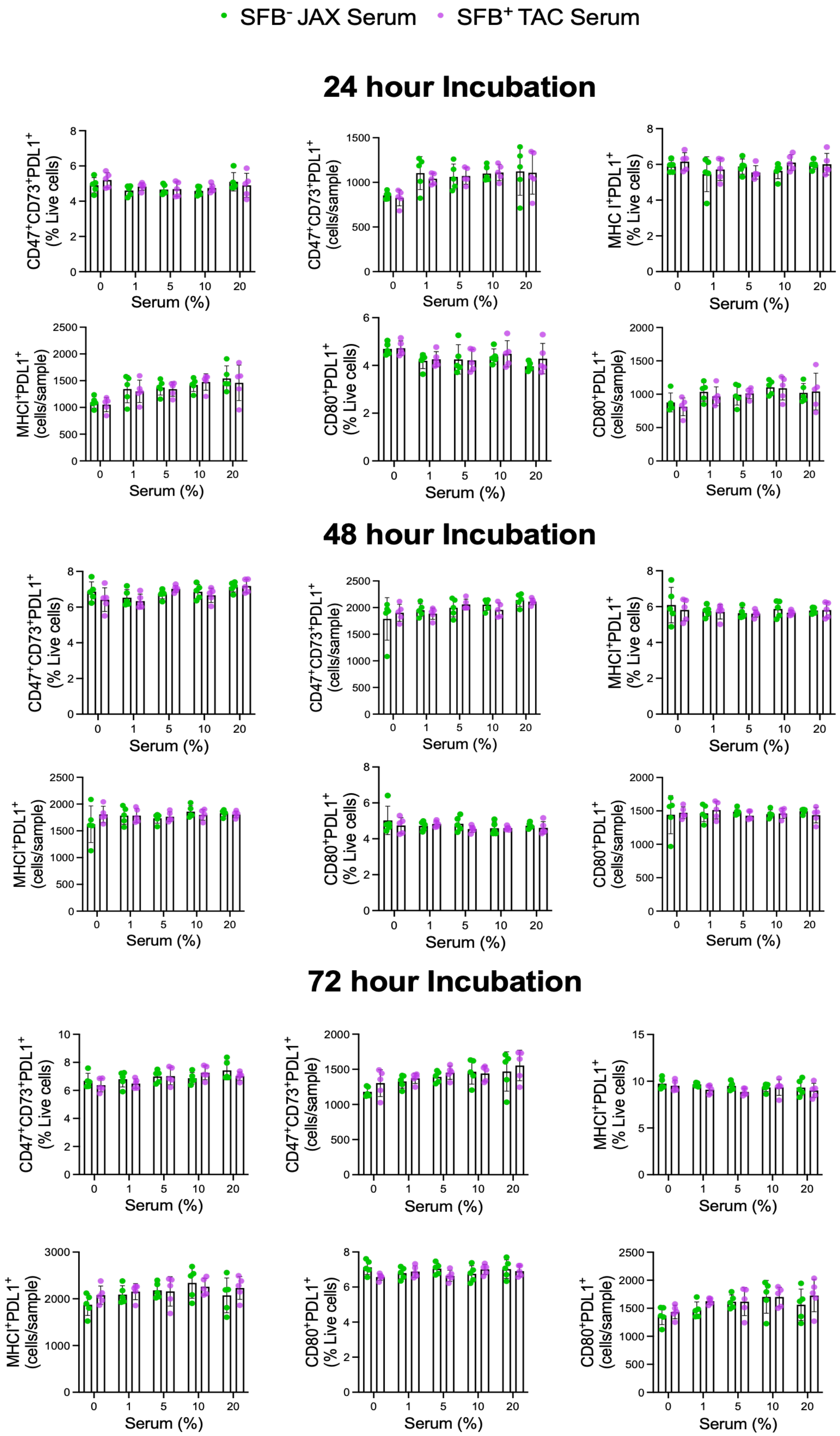

Supplemental Figure 10. Effects of serum from SFB⁺ or SFB⁻ mice on PyMT cell immune evasion properties. PyMT cells were treated with different concentrations of Serum (0, 0.1, 5, 10, or 20 %) and analyzed by flow cytometry after 24, 48, or 72 h of incubation. Cells were assessed for three immune-evasion-associated phenotypes: $CD47^+CD73^+PD\text{-}L1^+$ cells (immune-evasive phenotype), $MHC\text{-}I^+PD\text{-}L1^+$ cells (adaptive immune resistance phenotype), and $CD80^+PD\text{-}L1^+$ cells (checkpoint-costimulatory rheostat phenotype). n = 5 samples per condition. Data are expressed as Mean ± SD. Data were compared by two-way ANOVA and post hoc tests applying Bonferroni's correction for multiple comparisons. Non-significant comparisons not shown.

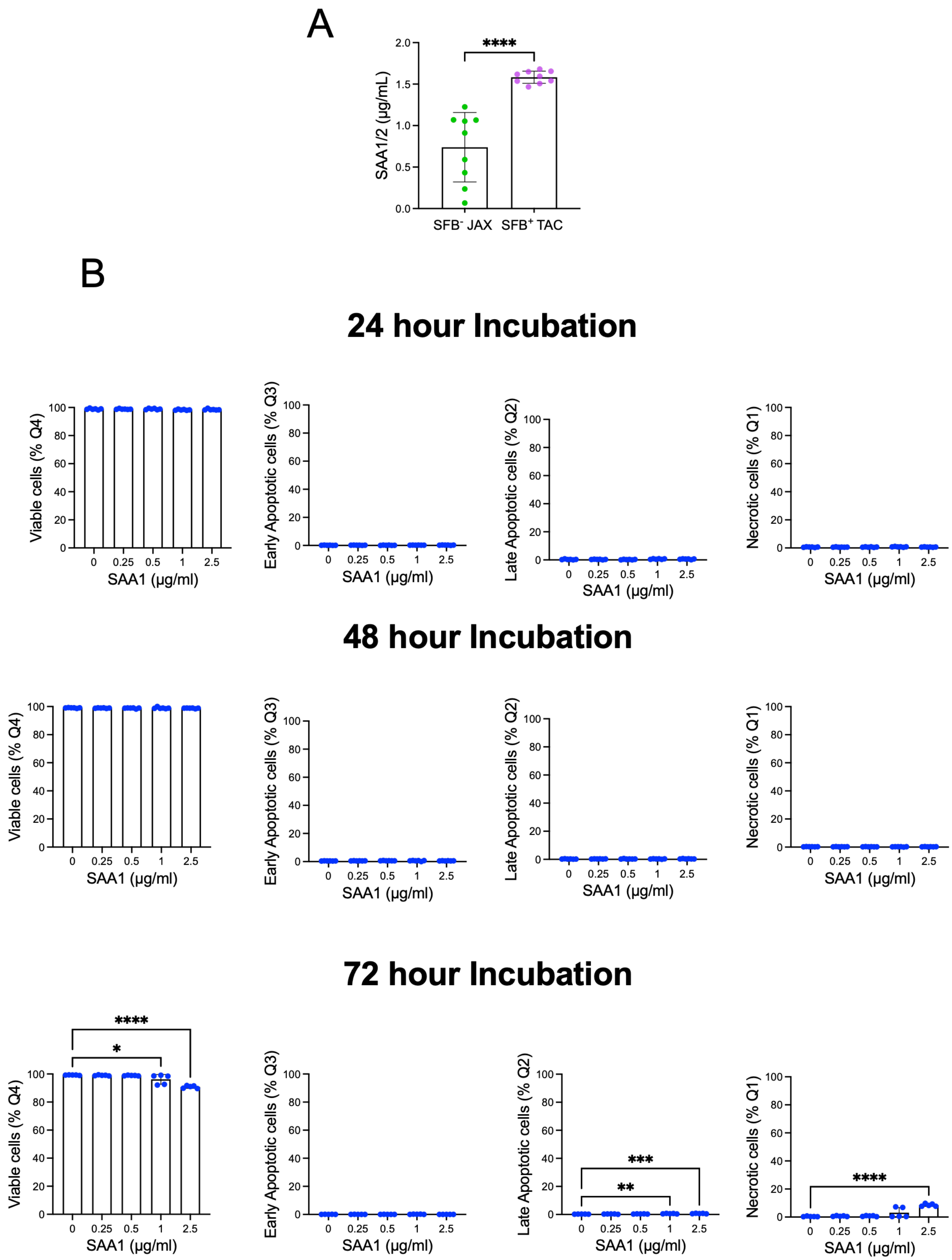

Supplemental Figure 11. Serum level of SAA1/2 and Effects of SAA1 on PyMT cell proliferation and survival. (A) Serum level of SAA1/2 in SFB$^+$ TAC mice and SFB$^-$ JAX mice. n = 9 samples per group. **(B)** PyMT cells were treated with different concentrations of SAA1 (0, 0.25, 0.5, 1, or 2.5 μg/ml) and analyzed using flow cytometry after staining with Annexin V-FITC and PI at 24 h, 48 h, and 72 h incubation. Four populations were indicated as necrotic dead cells (Q1), late apoptotic cells (Q2), early apoptotic cells (Q3), and viable cells (Q4). n = 5-6 samples per group. Data are expressed as Mean ± SD. * = P < 0.05, ** = P < 0.01, and **** = P < 0.0001 compared to indicated group. Data were compared by one-way ANOVA and post hoc tests applying Bonferroni's correction for multiple comparisons. Non-significant comparisons not shown.

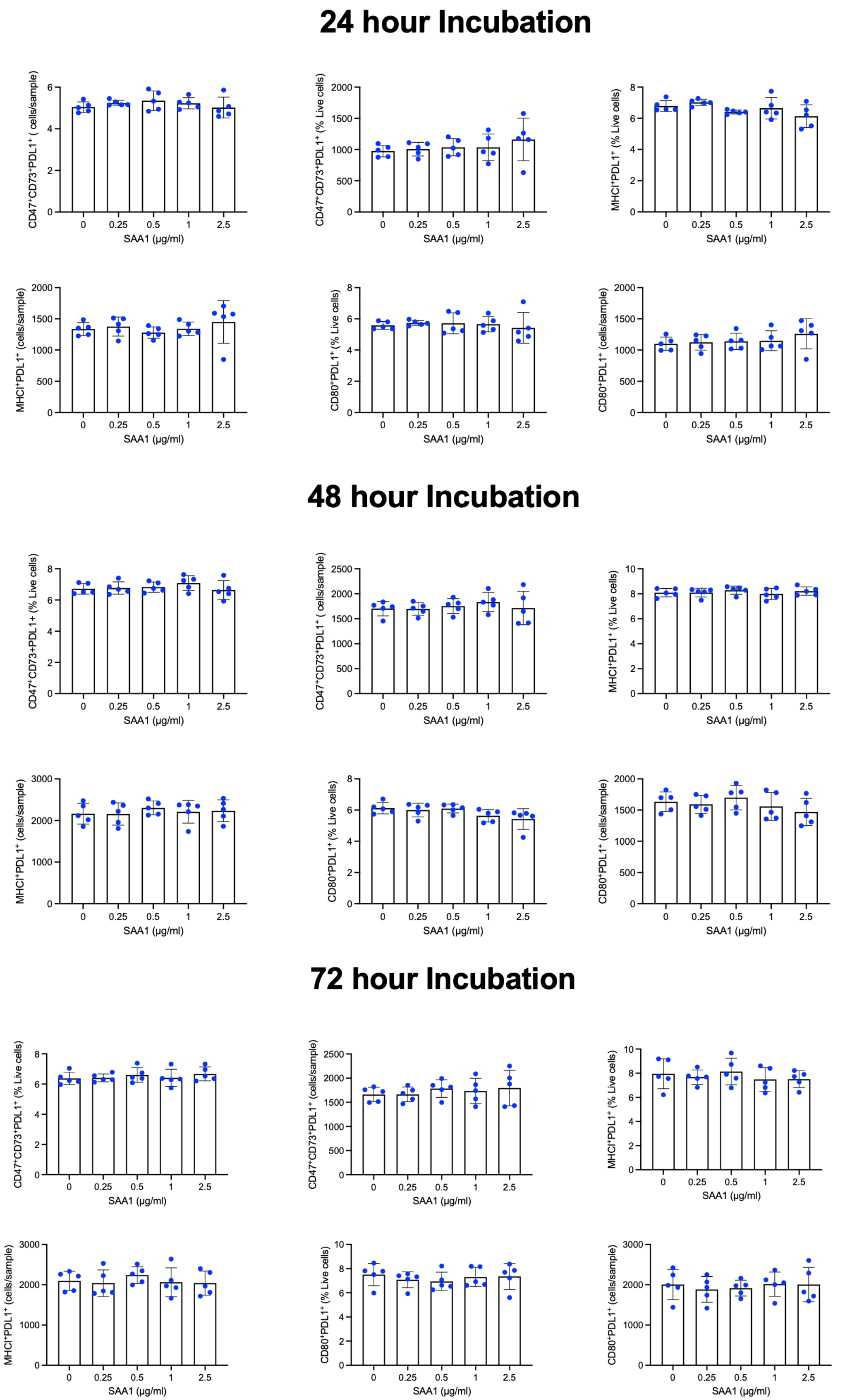

Supplemental Figure 12. Effects of SAA1 on PyMT cell immune evasion properties. PyMT cells were treated with different concentrations of SAA1 (0, 0.25, 0.5, 1, or 2.5 μg/ml) and analyzed by flow cytometry after 24, 48, or 72 h of incubation. Cells were assessed for three immune-evasion-associated phenotypes: $CD47^{+}CD73^{+}PD\text{-}L1^{+}$ cells (immune-evasive phenotype), $MHC\text{-}I^{+}PD\text{-}L1^{+}$ cells (adaptive immune resistance phenotype), and $CD80^{+}PD\text{-}L1^{+}$ cells (checkpoint-costimulatory rheostat phenotype). n = 5 samples per condition. Data are expressed as Mean ± SD. Data were compared by two-way ANOVA and post hoc tests applying Bonferroni's correction for multiple comparisons. Non-significant comparisons not shown.

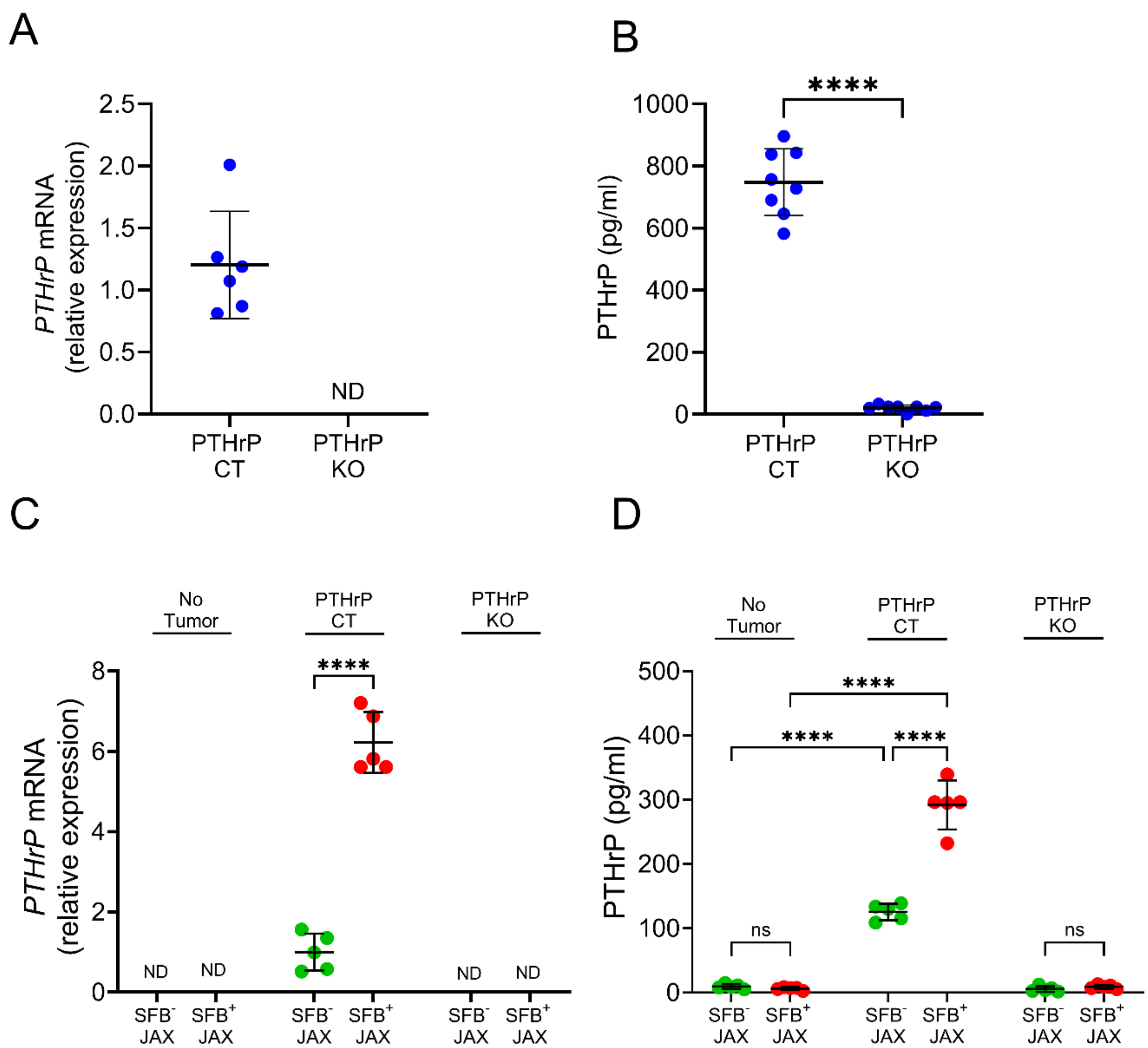

Supplemental Figure 13. PTHrP transcript and protein level in CT and KO PyMT cells. (A) PTHrP mRNA expression in CT and PTHrP KO PyMT cells. **(B)** PTHrP protein levels in conditioned media of CT and PTHrP KO PyMT cells. **(C)** PTHrP mRNA expression in tumor tissues of SFB^+ and SFB^- JAX mice injected with CT and PTHrP KO PyMT cells. **(D)** PTHrP protein levels in tumor tissues of SFB^+ and SFB^- JAX mice injected with CT and PTHrP KO PyMT tumor cells. Panels A,B: n = 4-8 per group. Panels C,D: n = 5 mice per group. Data are expressed as Mean ± SD. Normally distributed data were analyzed by 2-tailed t tests or two-way ANOVA and post hoc tests applying Bonferroni's correction for multiple comparisons.- ND = not detectable, ns = not significant, **** = p < 0.0001 compared with the indicated group.

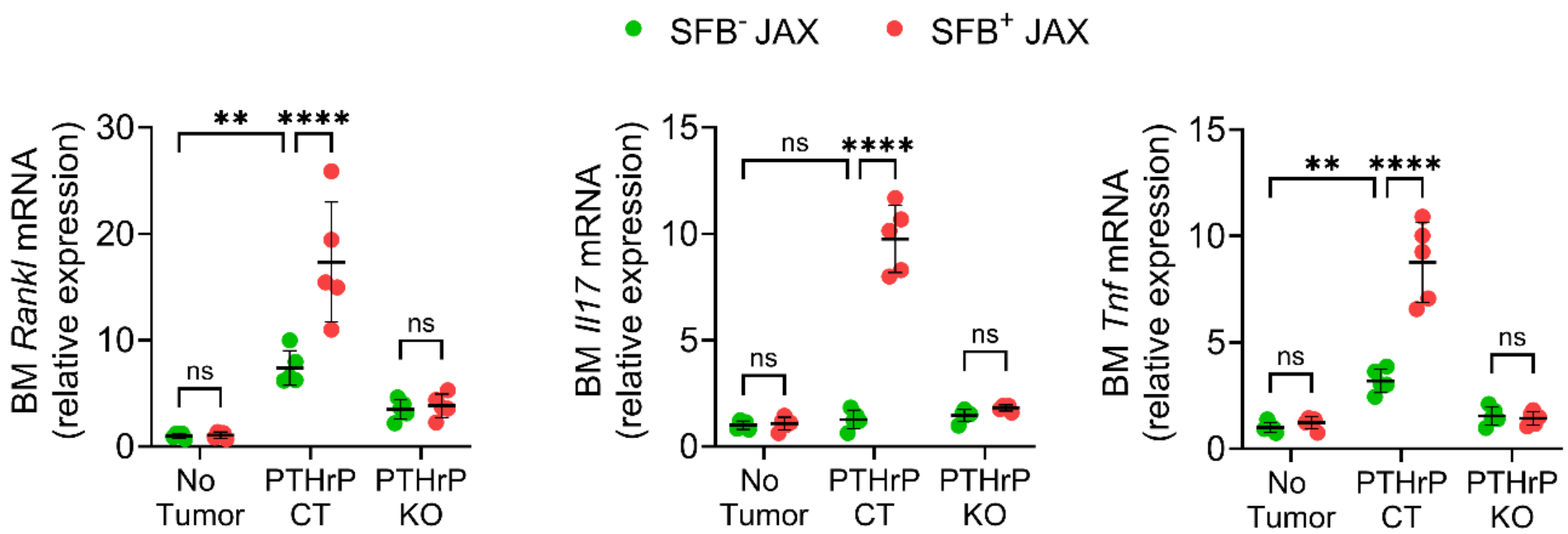

Supplemental Figure 14. Tumor site BM *Rankl*, *Il17*, and *Tnf* mRNA transcript levels in SFB^- JAX and SFB^+ JAX mice injected with CT or PTHrP KO PyMT breast cancer cells. n = 5 mice per group. Data are expressed as mean ± SD. All data were normally distributed and were analyzed by two-way ANOVA and post hoc tests applying Bonferroni's correction for multiple comparisons. ns = not significant, ** = P < 0.01, **** = P < 0.0001 compared with the indicated group.

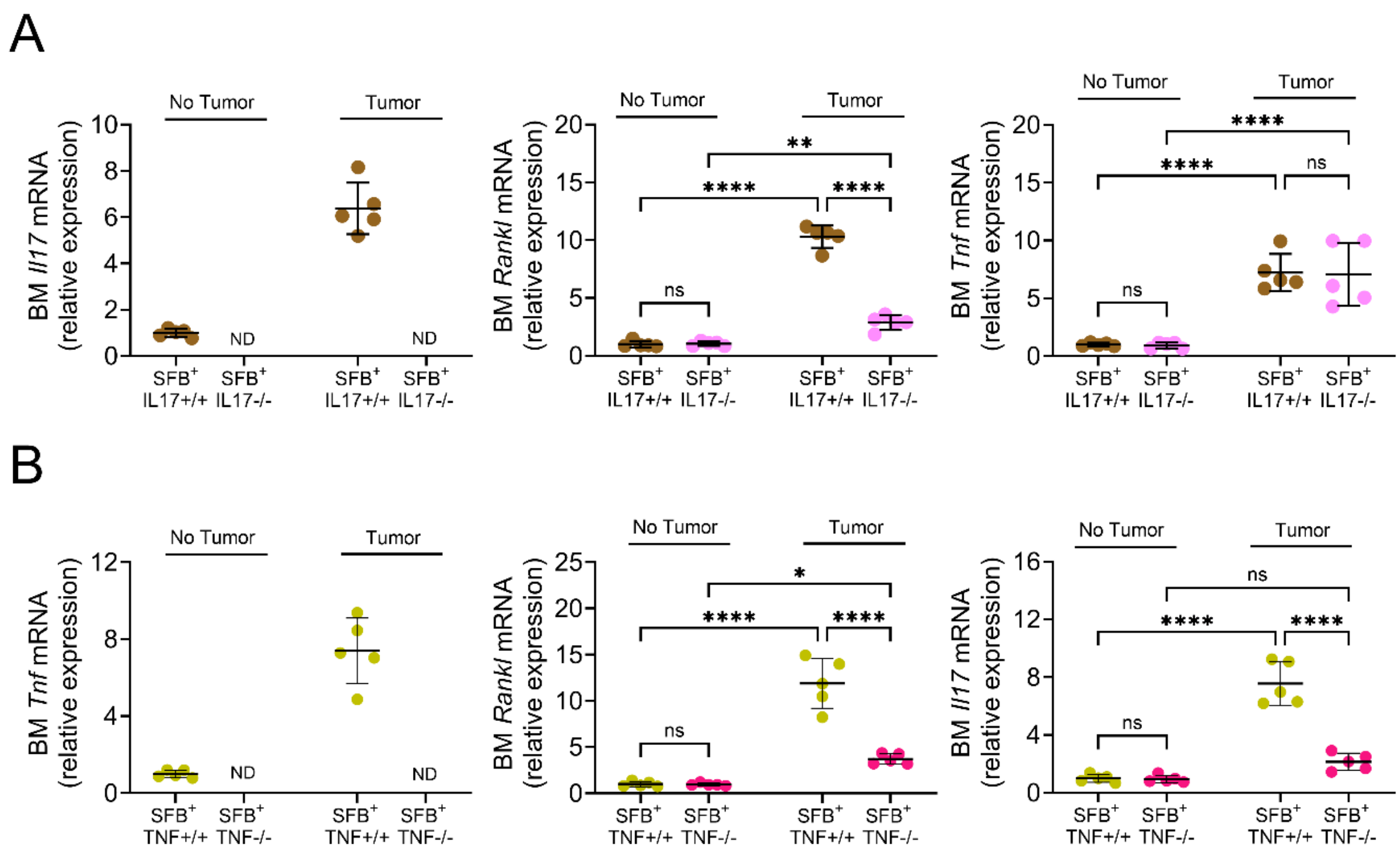

Supplemental Figure 15. Effects of IL17 and TNF deletion on the tumor site BM expression of *Rankl*, *Il17*, and *Tnf*. (A) *Rankl*, *Il17*, and *Tnf* mRNA expression at tumor site BM of IL17-/- mice. **(B)** *Rankl*, *Il17*, and *Tnf* mRNA expression at tumor site BM of TNF-/- mice. n = 5 mice per group. Data are expressed as mean ± SD. Normally distributed were analyzed by two-way ANOVA and post hoc tests applying Bonferroni's correction for multiple comparisons. ND = non detectable, ns = not significant, * = P < 0.05, *** = P < 0.001, **** = P < 0.0001 compared with the indicated group.
